# Optimal exercise modality and dose to improve depression in children and adolescents: a systematic review and Bayesian network meta-analysis

**DOI:** 10.3389/fpsyg.2026.1847609

**Published:** 2026-05-25

**Authors:** Jinping Hu, Dandan Li, Yan Zhao, Yiran Deng

**Affiliations:** 1 School of Education and Psychology, University of Jinan, Jinan, China; 2 School of Physical Education, Shandong University, Jinan, China

**Keywords:** childhood and adolescence, depression, dose–response, exercise intervention, meta-analysis, systematic evaluation

## Abstract

**Objectives:**

To determine the comparative efficacy of different exercise interventions on depressive symptoms in children and adolescents and to explore the precise non-linear dose–response relationship using Bayesian model-based network meta-analysis.

**Methods:**

A systematic search of four major databases (PubMed, Web of Science, PsycINFO, and Scopus) was conducted up to October 2024 for randomized controlled trials (RCTs). We employed pairwise, network, and dose–response meta-analyses to synthesize evidence regarding exercise modalities and dosage. Risk of bias was assessed using the Cochrane tool.

**Results:**

A total of 26 RCTs involving 3,507 participants were included. Network meta-analysis revealed that Group Training (SMD = −1.58, 95% CI: −2.54 to −0.62) and Aerobic Exercise (SMD = −0.60, 95% CI: −1.10 to −0.10) were effective interventions for alleviating depressive symptoms. In contrast, no statistically significant effects were observed for Mixed Training or Mind–Body Exercise, and the evidence for these modalities remained inconclusive. Dose–response modeling indicated a nonlinear curvilinear pattern rather than evidence of symptom worsening at higher doses. The minimum dose associated with a statistically significant effect was 360 METs-min/week, the estimated peak benefit occurred at 660 METs-min/week, and the effect attenuated and became statistically uncertain beyond 980 METs-min/week.

**Conclusion:**

Group Training and Aerobic Exercise are the optimal intervention modalities, demonstrating high therapeutic potency and intervention efficiency, respectively. The identified therapeutic window (360–980 METs-min/week) supports a “low-dose initiation and moderation” prescription strategy. However, due to the heterogeneity of the included studies, these findings should be interpreted with caution, and future high-quality trials are warranted.

**Systematic review registration:**

https://www.crd.york.ac.uk/prospero/display_record.php?ID=CRD42024596771, Registration number: CRD42024596771.

## 1 Introduction

Child and adolescent mental health has become a critical global public health concern. As a classic form of internalizing behavior within Emotional and Behavioral Disorders, depression is the primary cause of functional impairment and psychological distress in this population, often accompanied by recurrent symptoms such as reduced attention, severe guilt, and suicidal ideation (Centers for Disease Control and Prevention, 2026). Epidemiological data indicate that approximately 14% of adolescents meet the diagnostic criteria for depression before adulthood, with up to 25% experiencing a depressive episode (Merikangas et al., 2010; Hetrick et al., 2021). Furthermore, the global detection rate for adolescent depression is on an upward trend (Shorey et al., 2022). Early-onset depression is not limited to immediate symptoms; it disrupts key neurodevelopmental processes, including prefrontal cortex maturation and synaptic pruning, thereby increasing vulnerability to future stressors (Amos et al., 1997). Evidence confirms that about 67% of affected adolescents progress to severe psychiatric disorders in adulthood (Klein et al., 2009), leading to profound social and educational impairment (Copeland et al., 2021). These findings establish depressive symptoms in youth as a potent predictor of life-long mental health trajectory (Keenan et al., 2008). Therefore, implementing early interventions during this critical developmental period is paramount to restoring a healthy psychological trajectory.

Current clinical guidelines predominantly recommend psychotherapy and/or pharmacotherapy as first-line treatments for depression (MacQueen et al., 2016). However, both modalities face multiple barriers in real-world application. Psychotherapy often has a protracted onset of effect and demands high levels of abstract thinking and introspective capacity, posing significant challenges for adolescents with developing cognitive abilities (Garber et al., 2016). Furthermore, the scarcity of specialized personnel and high economic costs substantially restrict treatment accessibility (Radez et al., 2021). Meanwhile, pharmacotherapy in adolescents remains controversial due to its limited efficacy and potential risks, such as increased suicidal ideation, which compromises treatment adherence (Dwyer and Bloch, 2019). Compounded by illness stigma, resource disparity, and lack of family support, only 34% of community-dwelling affected youth receive professional help (Avenevoli et al., 2015), leaving up to 80% of patients facing a critical “treatment gap” (Kataoka et al., 2002). This context underscores the urgency of exploring adjunctive strategies that are simple to implement, have minimal side effects, and offer high sustainability.

Compared to traditional treatments, exercise intervention is viewed as a more sustainable and promising alternative due to its low cost and high adherence (Gråstén, 2017; Weeks et al., 2017). The antidepressant mechanisms of exercise are multifaceted (Peluso and de Andrade, 2005). Biologically, regular physical activity elevates Brain-Derived Neurotrophic Factor levels and modulates the Hypothalamic–Pituitary–Adrenal axis, thereby enhancing neuroplasticity and mitigating hormonal imbalances (Gujral et al., 2017; Kandola et al., 2019). Psychologically, exercise boosts self-efficacy and serves as an active distraction to interrupt ruminative thinking (Recchia et al., 2023). Socially, particularly through Group Training, it fosters social connection and belonging, which functions as a positive compensatory mechanism against social withdrawal (Harvey et al., 2018).

Despite the consensus on the efficacy of exercise, clear clinical guidance on the “optimal exercise prescription” remains elusive. Previous meta-analyses have largely focused on comparing discrete exercise modalities, leading to conflicting conclusions on which type is superior. For instance, one review reported that aerobic exercise, group training, resistance training, and mixed exercise were all effective in alleviating depressive symptoms in youth, with aerobic exercise showing the strongest effect, followed by group training (Li et al., 2023). Conversely, another study suggested that mind–body exercise (such as yoga) held advantages for emotional regulation (Bailey et al., 2018). Furthermore, systematic reviews emphasize that group activities, due to their inherent social support component, may be more beneficial than individual exercise modalities (Radovic et al., 2017). This overemphasis on modality overlooks a fundamental fact: intervention effectiveness ultimately depends on whether the”dose” has reached an effective level. Exercise dose, fundamentally a composite of the FITT principle (Frequency, Intensity, Time, and Type), constitutes a critical factor that directly influences intervention outcomes (Ferguson, 2014). The Bull et al. (2020) and the evidence-based report by the Physical Activity Guidelines Advisory Committee (Erickson et al., 2019) both highlight the dose–response relationship as a key element to prioritize in the formulation of exercise prescriptions for depression. Therefore, a systematic investigation into the synergistic effects of exercise modality and dose is crucial for enhancing the precision of exercise-based interventions.

The objective of this study is to systematically evaluate the relative efficacy of four exercise modalities (aerobic exercise, group training, mixed training, and mind–body exercise) using data from Randomized Controlled Trials. Building on this, we introduce the Bayesian Model-Based Network Meta-Analysis to assess the dose–response relationship of exercise, estimate the clinical effective dose range for improving depressive symptoms, and thus provide evidence-based, actionable guidelines for clinical prescription.

## 2 Methods

This study was conducted in accordance with the Cochrane Collaboration Handbook and the Preferred Reporting Items for Systematic Reviews and Meta-Analyses (PRISMA) extension statement for systematic reviews incorporating Network Meta-Analyses (NMAs) for health care (Hutton et al., 2015; Higgins et al., 2019). The study protocol was prospectively registered on the PROSPERO platform (registration number: CRD42024596771). Moreover, as all analyses were based on published literature, ethical review and informed consent from patients were not required for this study.

### 2.1 Search strategy

To determine the effects of different types of exercise on depressive symptoms in children and adolescents, we conducted a systematic search of four databases: PubMed, Web of Science, Scopus, and PsycINFO (search period: from inception to October 2024). The search strategy combined Medical Subject Headings (MeSH) and free-text terms, connected using Boolean operators “OR” “AND,” without any restrictions (e.g., language, region). The specific search strategies for each database are outlined in Supplementary file 1. Additionally, we reviewed previously published systematic reviews and meta-analyses on exercise interventions for depressive symptoms in children and adolescents to ensure no potentially valuable literature was missed.

### 2.2 Eligibility criteria

The inclusion criteria for literature followed the PICOS (Population / Intervention / Comparison / Outcome / Study design) principle. The study population was limited to children and adolescents without pre-determined health or psychological conditions, to ensure the inclusion of potentially valuable data and maintain sample representativeness. The specific inclusion and exclusion criteria are detailed in Supplementary file 4.1.

### 2.3 Study selection

Based on the inclusion and exclusion criteria, two researchers (D. D. Li and Y. Zhao) removed duplicate articles and screened study titles and abstracts. Subsequently, they reviewed the full texts to assess the eligibility of the remaining articles, ensuring that only studies meeting the criteria were included. In cases of disagreement between the two researchers during the screening process, a third experienced researcher (J. P. Hu) was consulted to resolve the discrepancy.

### 2.4 Data extraction and coding management

Data extraction was independently performed by two researchers (D. D. Li and Y. Zhao). The extracted information included authors, publication year, number of subjects, age, gender, and other demographic information, as well as exercise intervention details (modalities, duration, frequency, cycle), and outcome measurement tools. When data were unavailable or incomplete, attempts were made to contact the corresponding authors of the articles for assistance. The detailed process of data extraction and coding management is provided in Supplementary file 4.2.

### 2.5 Primary outcome

The primary outcome was the mean change in depressive symptoms from baseline to the end of the intervention in the intervention and control groups. When change scores were not directly reported, they were derived from reported statistics, including standard errors, confidence intervals, and sample sizes, using established methods (Egger et al., 2003; Lipsey and Wilson, 1993). If multiple post-intervention time points were reported, only the data assessed at the end of the intervention were extracted.

### 2.6 Risk of Bias and quality of evidence

The quality assessment of the included literature was independently conducted by two researchers using the original Cochrane Risk of Bias tool (Higgins, 2008). We retained this tool rather than the updated RoB 2 tool because the assessment in this review was conducted at the study level and had already been integrated into the completed GRADE framework. This approach also maintained consistency with the evidence-certainty assessment across all included comparisons. The scoring criteria included random sequence generation (selection bias), allocation concealment (selection bias), blinding of participants and personnel (performance bias), blinding of outcome assessment (detection bias), incomplete outcome data (attrition bias), selective reporting (reporting bias), and other biases. The quality information of the included studies was imported into Review Manager 5.4 software to generate a risk of bias graph.

The GRADE evidence grading system was used to assess the quality of evidence for the ranking of treatment effects of different exercise modalities, considering study limitations, indirectness and transitivity, statistical heterogeneity and inconsistency, imprecision, and publication bias (Salanti et al., 2014). Each study was assumed to have the highest initial quality rating and was subsequently rated as high, moderate, low, or very low quality after assessing the aforementioned factors.

### 2.7 Statistical analysis

Pairwise meta-analyses were performed to estimate the standardized mean difference (SMD) in depression scores between exercise interventions and control conditions, as well as direct comparisons across exercise modalities where available. Conventional network meta-analysis and MBNMA were then used for complementary analytical purposes. The conventional network meta-analysis synthesized direct and indirect evidence to estimate the relative effects of different exercise modalities, thereby establishing the treatment-level comparative efficacy structure of the evidence network. To examine whether intervention effects varied according to exercise dose, Bayesian model-based network meta-analysis was conducted within the same evidence network. MBNMA extends the network meta-analysis framework by modeling the relationship between dose and treatment effect and is therefore appropriate for estimating dose–response patterns across connected intervention nodes (Lu and Ades, 2004; Mawdsley et al., 2016). In addition, network meta-regression analyses were performed to explore the influence of potential effect modifiers on intervention outcomes. The covariates included intervention period, intervention frequency, session duration, and percentage of female participants, which were entered into the model as continuous or proportional variables, as appropriate. These analyses were conducted to assess the impact of these factors on the relative effect estimates of different exercise modalities and to further examine the robustness of the main findings.

The detailed statistical analysis process is provided in Supplementary file 4.3. All statistical analyses were conducted using R software (version 4.3.3), except that risk-of-bias figures were generated using Review Manager 5.4. Pairwise meta-analyses were performed using the “meta” package, and frequentist network meta-analyses were conducted using the “netmeta” package. Bayesian model-based dose–response network meta-analyses were performed using the “MBNMAdose” package. We used the default prior specifications implemented in MBNMAdose and did not impose custom informative priors. The key assumptions of MBNMA, including network connectivity, consistency, and transitivity, were assessed before dose–response modeling. Candidate dose–response functions, including Emax, restricted cubic splines, quadratic, and other recommended models, were fitted and compared primarily using the Deviance Information Criterion (DIC), supplemented by between-study standard deviation, the number of model parameters, and residual deviance. The quadratic random-effects model showed the best overall fit and was therefore selected to estimate the nonlinear dose–response relationship. Model convergence and adequacy were assessed using standard diagnostic outputs, and full model-fit and diagnostic details are provided in Supplementary file 7.3.

## 3 Results

### 3.1 Literature retrieval and screening

**Figure 1:**
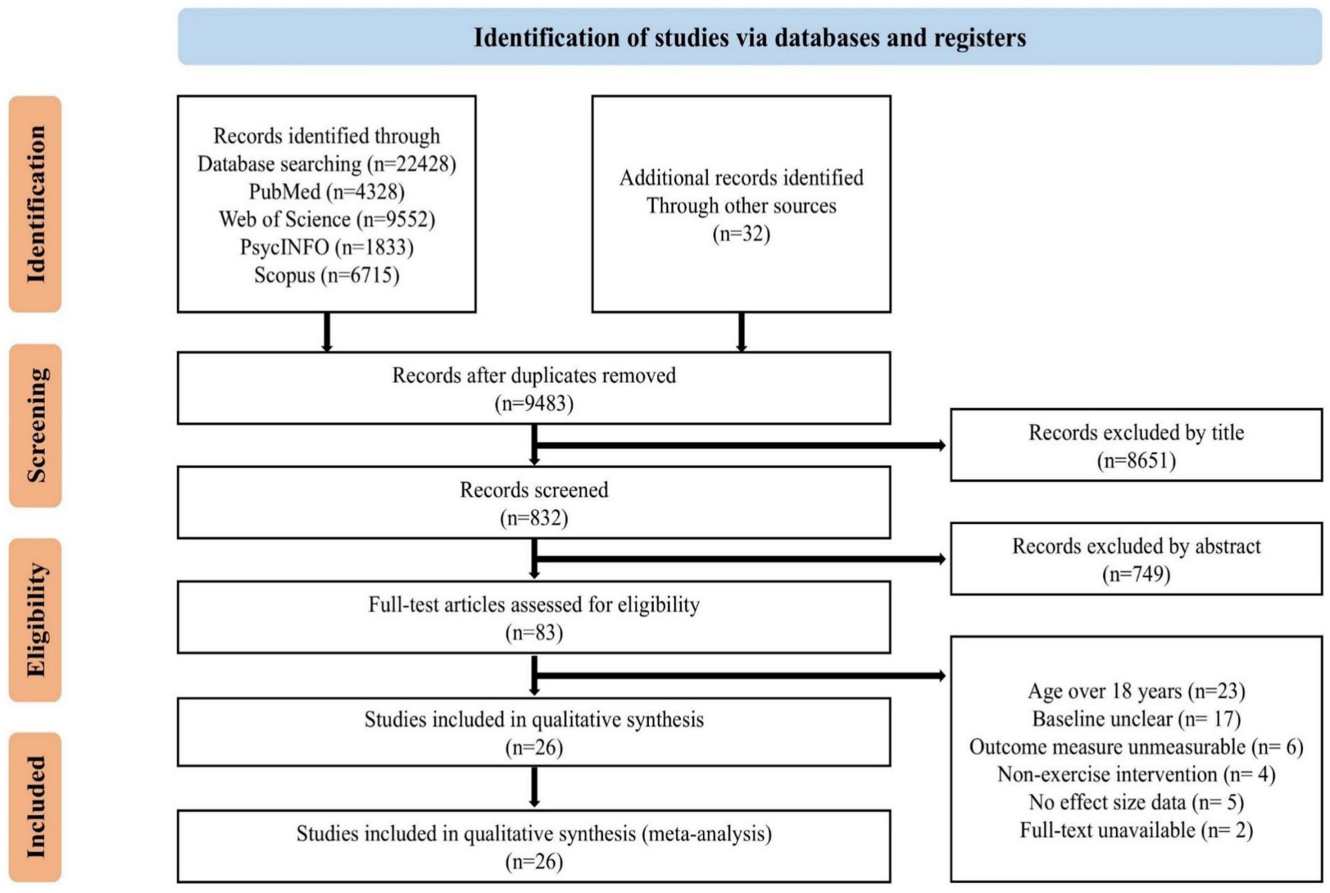
PRISMA flowchart of included studies.

A total of 22,428 records were retrieved from the original databases, and 32 records were retrieved from relevant systematic reviews and meta-analyses. After removing duplicates, a total of 9,483 records were screened. Based on the titles, 8,651 irrelevant studies were excluded, and subsequently, based on the abstracts, 749 irrelevant studies were further excluded. A total of 83 eligible full-text articles were selected, and ultimately, 26 studies were included in the analysis. The detailed process of literature retrieval and screening is shown in Figure 1. The paired comparison network diagram is provided in Supplementary file 6.1.

### 3.2 Characteristics of included studies

The total sample size of the included 26 studies was 3,507 participants, with a mean age ranging from 7 to 17 years. Among them, there were 1907 participants in the experimental group and 1,600 participants in the control group. Of the 26 studies, 4 were head-to-head comparisons, and 1 tested different doses of the same exercise modalities. The single duration of exercise interventions ranged from 8 to 90 min, the intervention period ranged from 6 to 208 weeks, and the intervention frequency ranged from 1 to 7 times per week. The exercise intensity matched the exercise type. The characteristic information of all studies is listed in Supplementary file 2.

### 3.3 Risk of bias and quality of evidence

Most studies had a low risk of bias in random sequence generation (21 studies), 1 study failed to implement allocation concealment, 8 studies explicitly stated the failure to achieve blinding of participants or assessors, 24 studies had data integrity, 23 studies had no selective reporting, and only 3 reported obvious risks of other biases. A detailed quality assessment report is provided in Supplementary file 3. Additionally, according to the GRADE evaluation criteria, the overall quality of the outcome indicators was low (Supplementary file 6.5).

### 3.4 Paired meta-analysis

The results of the paired meta-analysis demonstrated that both Group Training (GT) and Aerobic Exercise (AE) significantly reduced depressive symptoms in children and adolescents. GT exhibited the largest effect size, SMD = −1.58 (95% CI: −2.54 to −0.62), with zero heterogeneity (*I*^2^ = 0%). AE’s effect size was SMD = −0.60 (95% CI: −1.10 to −0.10), with moderate heterogeneity (*I*^2^ = 59%). In contrast, Mixed Training (MT) had an effect size of SMD = −0.32 (95% CI: −0.77 to 0.14, *I*^2^ = 0%), and Mind–Body Exercise (MBE) had SMD = 0.04 (95% CI: −0.69 to 0.77, *I*^2^ = 0%); neither intervention achieved statistical significance. The detailed results of the paired meta-analysis are presented in Supplementary file 5.1. Furthermore, the study used a corrected funnel plot to assess potential publication bias, and Egger’s test (*p* = 0.124) did not detect statistically significant funnel plot asymmetry. However, this result should be interpreted with caution, as the statistical power of Egger’s test may be limited given the number of included studies. Supplementary file 5.2 contains the corrected funnel plot and the results of Egger’s test.

### 3.5 Network meta-analysis

**Figure 2:**
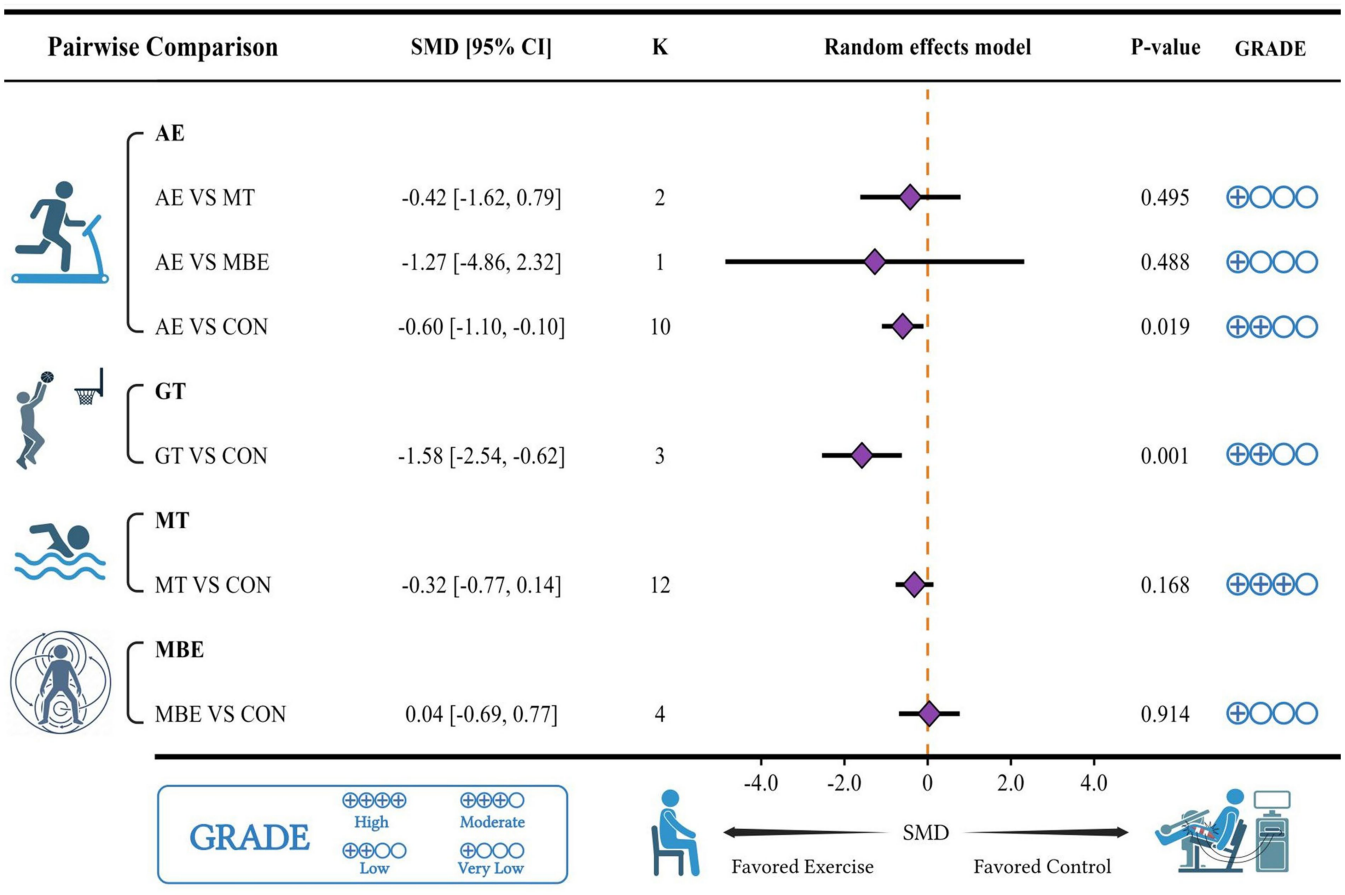
Pairwise comparison of all exercise interventions.

The Network Meta-Analysis further compared the relative efficacy of the four exercise modalities, and the results demonstrated that GT and AE exhibited a statistically significant advantage. The number of studies contributing to each node was as follows: Aerobic Exercise (*k* = 10), Group Training (*k* = 3), Mixed Training (*k* = 12), and Mind–Body Exercise (*k* = 4). Comparisons involving MT and MBE were characterized by substantial imprecision (e.g., MT vs. GT: SMD = −1.27, 95% CI: −4.86 to 2.32), suggesting that these estimates should be interpreted cautiously and may reflect limited precision rather than clear evidence of no effect. According to the P-score ranking, Group Training (*p* = 0.987)ranked first with the highest probability of being the best, followed by Aerobic Exercise (*p* = 0.701) in second place, while Mixed Training (*p* = 0.473) and Mind–Body Exercise (*p* = 0.176) ranked third and fourth, respectively (Figure 2; Table 1). The network model showed low between-study heterogeneity (τ^2^ = 0.0901; *Q* = 29.04, *df* = 25, *p* = 0.262; *I*^2^ = 13.9%). Assessment of inconsistency based on direct–indirect comparisons did not identify statistically significant inconsistency for estimable comparisons (Supplementary file 6.2). These findings suggest no clear evidence of important inconsistency, although the limited number of closed loops warrants cautious interpretation. Sensitivity analysis further confirmed that the ranking and effect sizes of the exercise interventions remained stable after excluding problematic literature, demonstrating high reliability (Supplementary file 6.4).

**Table 1:** Results of comparative effects of interventions from network meta-analysis and pairwise meta-analysis.

AE	NA	−0.42 (−1.62 to 0.79)	−1.27 (−4.86 to 2.32)	**−0.60 (−1.10 to −0.10)**
−0.96 (−2.03 to 0.11)	GT	NA	NA	**−1.58 (−2.52 to −0.62)**
−0.31 (−0.94 to 0.32)	**−1.27 (−2.32 to −0.21)**	MT	NA	−0.32 (−0.77 to 0.14)
−0.60 (−1.47 to 0.27)	**−1.64 (−2.82 to −0.44)**	−0.37 (−1.22 to 0.48)	MBE	0.04 (−0.69 to 0.77)
**−0.62 (−1.11 to −0.13)**	**−1.58 (−2.54 to −0.62)**	−0.31 (−0.78 to 0.13)	0.06 (−0.67 to 0.78)	CON

Results of the network meta-analyses are presented in orange, and results of the pairwise meta-analyses are presented in green. Blue cells indicate the intervention labels on the diagonal. Bold values indicate statistically significant comparisons. AE, aerobic exercise; GT, group training; MT, mixed training; MBE, mind–body exercise; NA, not available.

### 3.6 Dose–response relationship

**Figure 3:**
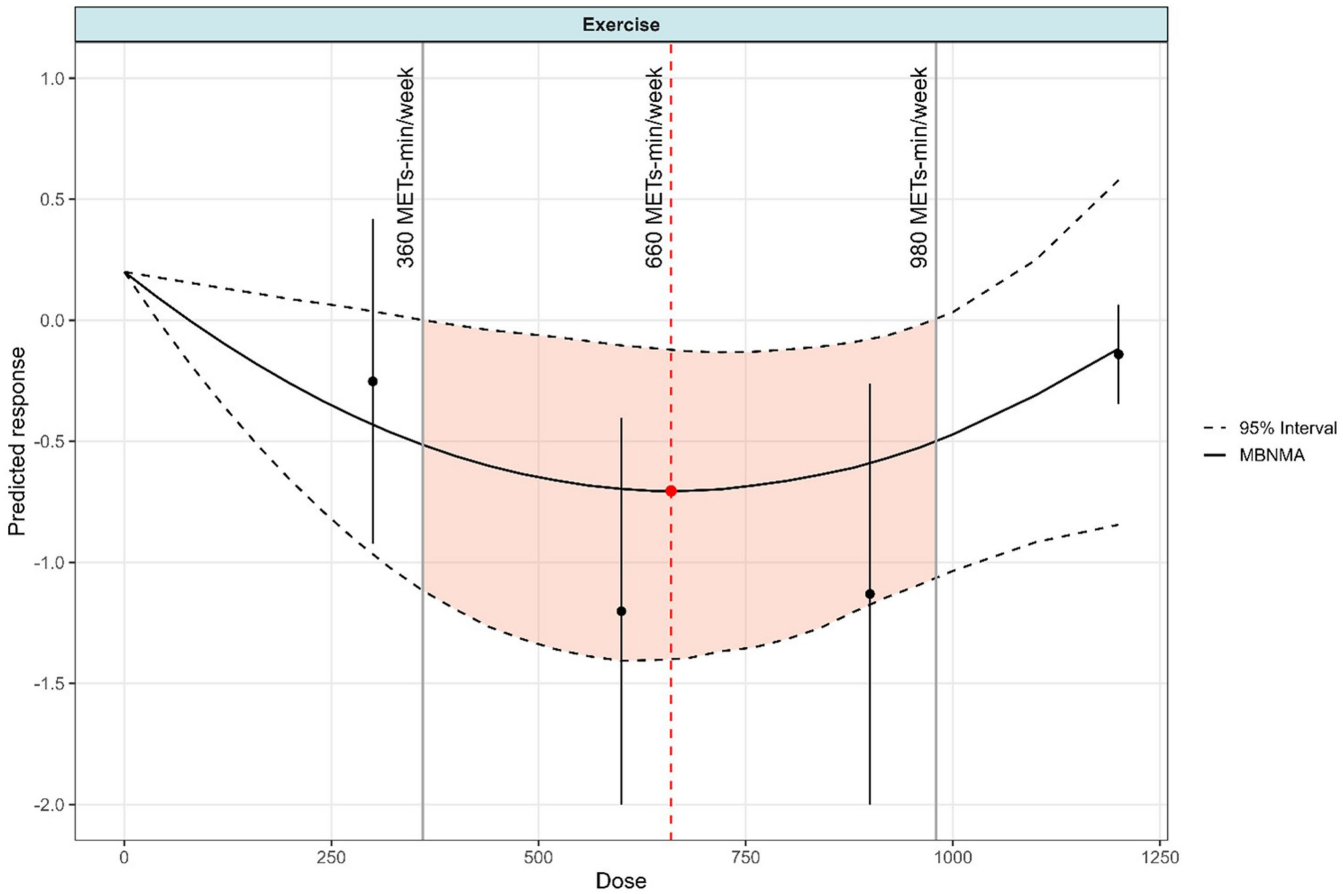
Dose–response relationship between total physical activity dose and changes in depressive symptoms in children and adolescents.

Figure 3 illustrates the dose–response relationship between total exercise volume and changes in depressive symptoms. The model suggested a nonlinear curvilinear pattern characterized by an initial increase in antidepressant benefit, an estimated peak effect at 660 METs-min/week, and attenuation of benefit at higher doses. The minimum dose associated with a statistically significant response was 360 METs-min/week, with an effect size of SMD = −0.53 (95% CrI: −1.09 to −0.01). The estimated peak benefit occurred at 660 METs-min/week, with an effect size of SMD = −0.73 (95% CrI: −1.38 to −0.11). The upper boundary at which the estimated effect remained statistically significant was 980 METs-min/week (SMD = −0.52, 95% CrI: −1.11 to −0.01). Importantly, this pattern should be interpreted as diminishing marginal benefit rather than evidence that higher exercise doses worsen depressive symptoms.

**Figure 4:**
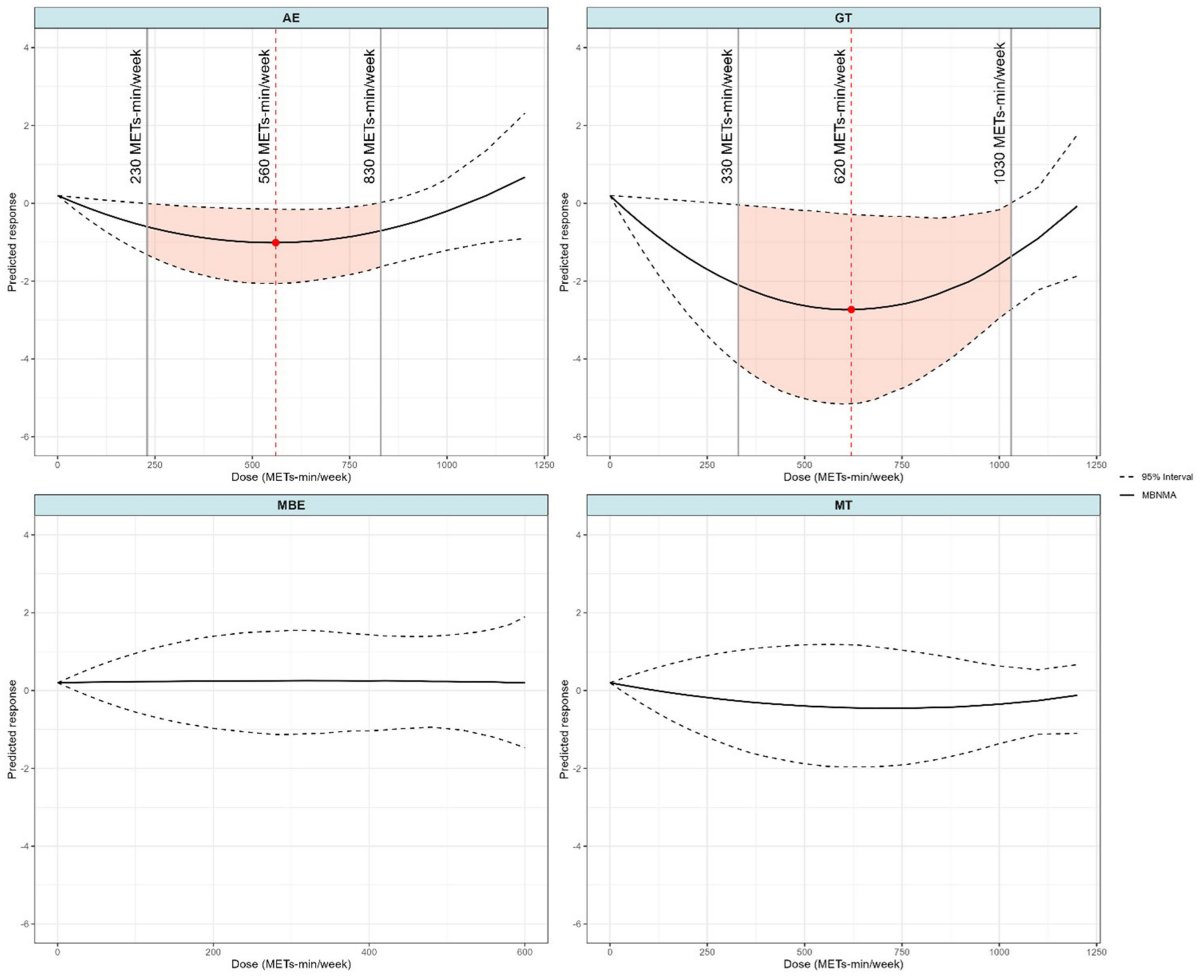
Dose–response relationships between different types of exercise and exercise dose and changes in depressive symptoms in children and adolescents.

Figure 4 illustrates the different dose–response patterns for the four exercise interventions in improving depressive symptoms. Nonlinear dose–response patterns were observed for both AE and GT, with each modality showing an estimated peak benefit followed by attenuation at higher doses. Specifically, AE’s minimum effective dose for significant clinical improvement was 230 METs-min (SMD = −0.62, 95%CrI: −1.32 to −0.01), with the effect peak appearing at 560 METs-min (SMD = −1.04, 95%CrI: −2.08 to −0.18). In contrast, GT required a higher dose to produce a significant effect, with its effective threshold at 330 METs-min (SMD = −2.10, 95%CrI: −4.31 to −0.01), and exhibited the strongest clinical benefit at 620 METs-min (SMD = −2.74, 95%CrI: −5.41 to −0.19).

### 3.7 Network meta-regression

**Figure 5:**
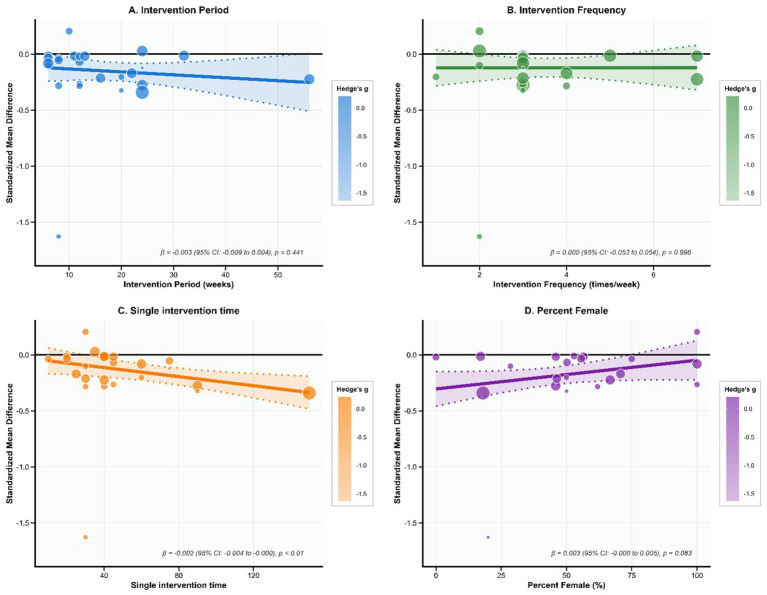
Network meta-regression. **(A)** Intervention period. **(B)** Intervention frequency. **(C)** Single intervention time. **(D)** Percent female.

Exploratory network meta-regression suggested a possible association between session duration and the antidepressant effect of exercise (Figure 5). Longer session duration was associated with a more negative SMD estimate (*β* = −0.002, 95% CI: −0.004 to −0.000, *p* < 0.050), indicating a possible relationship with greater symptom reduction. However, the magnitude of this coefficient was small and the statistical significance was marginal, and this result should be interpreted cautiously because of the limited number of included studies, the number of covariates examined, and the potential risk of overfitting. Therefore, this finding should be regarded as hypothesis-generating rather than confirmatory evidence that longer single-session exercise duration produces superior therapeutic effects.

Furthermore, the overall intervention period (*β* = −0.003, 95% CI: −0.009 to 0.004, *p* = 0.441) and intervention frequency (*β* = 0.000, 95% CI: −0.053 to 0.054, *p* = 0.996) did not show significant global moderating effects on the intervention outcomes (Figure 5). Additionally, the percentage of female participants did not significantly moderate the therapeutic effects (*β* = 0.003, 95% CI: −0.000 to 0.005, *p* = 0.083).

## 4 Discussion

### 4.1 Main findings

This study utilized Bayesian Network Meta-Analysis to not only confirm the significant efficacy of exercise intervention in alleviating depressive symptoms in children and adolescents, but also to precisely delineate the dose–response characteristics across different exercise modalities.

Firstly, we established the efficacy profile of each exercise modality. Results confirmed that after a minimum of 6 weeks of intervention, both Group Training and Aerobic Exercise demonstrated significant antidepressant effects, whereas Mixed Training and Mind–Body Exercise did not reach statistical significance. These non-significant findings should be interpreted cautiously, as they are more likely to reflect the current limits of the evidence than to indicate that their potential benefits can be ruled out. Secondly, the study revealed a nonlinear curvilinear dose–response pattern, in which antidepressant benefits increased up to an estimated peak and then attenuated at higher doses. The results indicated that 360 METs-min per week was the minimum effective threshold for inducing a significant antidepressant response. Furthermore, 660 METs-min was identified as the optimal treatment dose, while 980 METs-min represented the upper boundary at which the estimated effect remained statistically significant in the current model; estimates beyond this range suggested attenuated and less certain benefits rather than worsening outcomes. Lastly, the analysis highlighted distinct dose–response characteristics across the modalities. GT demonstrated the strongest antidepressant effect in terms of therapeutic potency, with its effect peak significantly surpassing other modalities. AE, however, exhibited a superior intervention efficiency, with its optimal dose threshold (560 METs-min) being substantially lower than that of GT and other interventions. Conversely, the dose–response curves for MT and MBE were relatively flat across all dose ranges, reflecting the difficulty of achieving stable, dose-dependent benefits with these two modalities under the current experimental settings. In summary, these quantified data provide the theoretical foundation for developing layered and precise exercise prescriptions.

### 4.2 Comparison with existing evidence

This study’s findings further confirm the universal value of exercise intervention in alleviating depressive symptoms in children and adolescents. Aerobic Exercise (AE), as a classic modality in antidepressant intervention, demonstrated a robust mitigating effect in this study, consistent with previous literature (Miller et al., 2020). The efficacy of AE is underpinned not only by its low cognitive threshold and minimal effective dose but also by a strong neurobiological foundation. Research indicates that depression is closely associated with reduced levels of Brain-Derived Neurotrophic Factor (BDNF) (Khan et al., 2018). Regular aerobic exercise has been proven to effectively upregulate BDNF levels in depressed individuals, directly improving symptoms by promoting neurogenesis and enhancing synaptic plasticity (Luo et al., 2019).

Simultaneously, the present findings underscore the superior standing of Group Training in depression intervention. Contrary to prior research that often focused solely on physiological stimuli like aerobic or resistance training, this study identified the potent symptomatic improvement effect of GT. This finding may be interpreted in light of the “Social Interaction Hypothesis”, suggesting that the social connection and sense of belonging provided by group exercise may function as a compensatory social mechanism that helps address the social withdrawal commonly observed in depression (Cohen and Wills, 1985; Bang et al., 2024; Klemmer et al., 2025). Recent meta-analyses similarly noted that group intervention demonstrated markedly superior efficacy over individual modalities, with this advantage attributed to the positive shaping of self-esteem and sense of belonging via social interaction (Yan et al., 2025). GT’s high ecological validity, which makes it more reflective of adolescents’ real-life social settings than single-mode exercise, suggests that shifting the intervention focus from mere physical exertion to structured social interaction is crucial for optimizing therapeutic outcomes. However, the large magnitude of GT’s effect (SMD = −1.58) warrants cautious interpretation. Despite its top probability ranking, the broad 95% Credible Interval (−2.54 to −0.62) implies considerable inter-study variability. Furthermore, the certainty of evidence remains low per the GRADE system, primarily due to methodological limitations like inadequate blinding and small sample sizes in the original studies. Consequently, while GT shows strong clinical potential, further high-quality, large-scale, head-to-head randomized controlled trials are needed to confirm its long-term stability across different depressive subtypes.

In contrast, Mixed Training did not demonstrate a statistically significant antidepressant effect in this study, which is broadly consistent with recent findings in children and adolescents (Zhou et al., 2025). However, given the imprecision of the corresponding estimates, this result is better interpreted as inconclusive rather than as evidence of no effect. From a sport physiology perspective, this non-significant result may be a consequence of stimulus intensity dispersion. In MT protocols, the load and volume allocated to a single stimulus (e.g., aerobic or resistance) might be insufficient to reach the physiological threshold required to induce antidepressant neuroadaptation, which may consequently limit the clinical efficacy of the mixed modality in this specific adolescent population. Furthermore, from a methodological standpoint, the limited number of MT studies and the high heterogeneity of their intervention protocols suggest that insufficient statistical power may have obscured any actual effect.

Similarly, Mind–Body Exercise did not show a statistically significant improvement in depressive symptoms in the present analysis. Given the small number of contributing studies and the wide confidence intervals, this result is better interpreted as inconclusive rather than as evidence of no effect. Several factors may help explain this pattern, including both psychological and physiological considerations. First, MBE often requires sustained attention, introspection, and active emotional regulation, and common features of depression in youth, such as attentional difficulties and negative self-cognition, may reduce engagement with these regulatory processes (Bourke et al., 2023). Second, from a physiological perspective, the relatively low exercise load of many MBE programs may be insufficient to induce the neurobiological changes associated with stronger antidepressant effects, thereby limiting their observable clinical impact (Li et al., 2024).

The nonlinear dose–response pattern identified in this study helped delineate a tentative dose range associated with antidepressant benefit in adolescents. The model estimated the largest benefit at 660 METs-min/week, whereas estimates above approximately 980 METs-min/week were weaker and less precise. This attenuation should be interpreted as diminishing marginal benefit rather than evidence that higher exercise doses worsen depressive symptoms. Compared with the more linear patterns often reported in adult literature (Leung et al., 2026; Noetel et al., 2024), this finding may be consistent with age-related differences in dose tolerance, although the present aggregate data cannot directly test developmental mechanisms. Potential explanations include differences in physiological tolerance, school schedules, adherence, perceived exertion, and contextual burden, all of which may limit the acceptability or sustained implementation of higher exercise doses in adolescents. These explanations should be regarded as hypothesis-generating and require future trials that directly measure adherence, perceived exertion, stress physiology, and long-term outcomes.

### 4.3 Clinical implications

The findings of this study provide a preliminary quantitative reference for exercise interventions targeting depression in children and adolescents. First, the results suggest that both Aerobic Exercise (AE) and Group Training (GT) may be considered preferential modalities for alleviating depressive symptoms. AE, given its lower effective dose and relatively simple cognitive demands, may be more suitable as an initial option for children with more severe depressive symptoms or marked social avoidance. In contrast, GT, with its higher estimated efficacy and inherent social component, may offer additional value for improving social competence and maximizing clinical benefit in appropriate cases. However, these suggestions should be interpreted cautiously, as the current evidence does not yet support stratified recommendations based on depression severity, social avoidance, or intervention sequence.

Second, the therapeutic window identified in this study (360–980 METs-min/week) may provide an evidence-informed framework for dose adjustment. The optimal estimated dose was 660 METs-min/week. In clinical or rehabilitation settings, this value may serve as a preliminary reference point, while 360 METs-min/week may be considered a lower starting point for gradual adjustment according to individual preference, tolerance, and clinical presentation. These dose-related implications should be regarded as preliminary rather than as definitive prescription thresholds.

Third, the findings also suggest that Mixed Training may still have practical value as a supplementary format. Although MT did not demonstrate a statistically significant dose–response relationship, its negative nonlinear trend indicates that combining exercise modalities may help diversify intervention delivery, reduce monotony, and potentially support long-term adherence (Eime et al., 2013), even though its antidepressant efficacy remains uncertain.

Finally, the currently non-significant findings for MT and MBE should not be misinterpreted as evidence of inefficacy. Rather, the available evidence for these modalities remains limited and uncertain because of the wide confidence intervals surrounding the current estimates. Clinicians should therefore interpret the current preference for AE and GT as a recommendation based on relative evidence strength, rather than as an exclusion of other potentially beneficial modalities. Overall, these clinical implications should be interpreted as preliminary and hypothesis-generating, pending validation in higher-quality and more targeted trials.

### 4.4 Limitations

Several limitations of this study should be acknowledged. First, the certainty of evidence was constrained by the methodological quality of the original studies. Most trials provided insufficient reporting on allocation concealment and blinding procedures, and their reliance on self-report scales may have introduced measurement bias. Together, these issues contributed to lower GRADE ratings for some comparisons. Future RCTs should adhere more closely to CONSORT guidelines and incorporate blinded outcome assessment to strengthen internal validity. Second, heterogeneity was present in the standardization and reporting of exercise dose. Although this study harmonized MET-min calculations, differences in intensity monitoring (e.g., heart rate), adherence reporting, and dose classification across the original studies may have introduced estimation bias. In addition, although the treatment-level and dose-level evidence networks were connected, the total number of included trials was modest, and some intervention nodes and dose ranges were supported by limited evidence. Accordingly, the comparative and dose–response estimates, particularly those involving sparsely populated nodes or specific dose ranges, should be interpreted as preliminary and require confirmation in larger head-to-head and dose-ranging trials. Third, the lack of long-term follow-up limited the evaluation of sustained efficacy. Most studies reported only immediate post-intervention effects and lacked follow-up data beyond 6 months, making it difficult to determine whether exercise-induced neurobiological adaptations are maintained over time. Future studies should incorporate follow-up assessments to clarify both detraining effects and the long-term maintenance of intervention benefits. Fourth, the network meta-regression analyses should be interpreted as exploratory. Given that only 26 trials were included while multiple study-level covariates were examined, these analyses may have been underpowered and vulnerable to unstable estimates and potential overfitting. Therefore, the association between session duration and depressive symptom reduction should be regarded as hypothesis-generating and requires confirmation in larger evidence networks or individual participant data. Fifth, external validity was limited by participant characteristics. The included populations consisted primarily of adolescents with subthreshold symptoms or mild-to-moderate depression, whereas clinically diagnosed inpatient samples with severe depression were scarce. Accordingly, caution is warranted when generalizing these findings to adolescents with severe clinical depression. In addition, although no explicit language restriction was imposed during screening, the literature search was conducted primarily in major English-language databases. Consequently, potentially relevant studies published in Chinese or other non-English languages may not have been fully captured, which may have introduced language bias and reduced the comprehensiveness of the evidence base.

## 5 Conclusion

This study employed a Bayesian network meta-analysis to systematically quantify the antidepressant effects of exercise intervention and its dose–response characteristics in children and adolescents. The findings establish that Group Training and Aerobic Exercise are the optimal intervention modalities for alleviating depressive symptoms, demonstrating distinct advantages in therapeutic potency and intervention efficiency, respectively. Furthermore, the dose–response analysis revealed a nonlinear curvilinear pattern, whereby clinical benefits emerged at approximately 360 METs-min/week, reached an estimated peak at 660 METs-min/week, and attenuated beyond 980 METs-min/week without providing evidence of symptom worsening at higher doses. Given the limited certainty of evidence and individual heterogeneity, clinical application warrants caution, and a principle of low-dose initiation and moderation is recommended. Future high-quality, head-to-head RCTs are urgently needed to confirm the long-term efficacy and optimal dose windows for different modalities, thereby facilitating the development of standardized guidelines.

## Data Availability

The original contributions presented in the study are included in the article/Supplementary material, further inquiries can be directed to the corresponding author.
