# Supplementary material for: Optimal exercise modality and dose to improve depression in children and adolescents: a systematic review and Bayesian network meta-analysis
Source: Front Psychol. 2026 May 25;17:1847609. doi: 10.3389/fpsyg.2026.1847609 (PMC13243028; doi:10.3389/fpsyg.2026.1847609)
Supplement: Supplementary file 1 [file Supplementary_file_1.docx]

**Optimal exercise modality and dose to improve depression in children and adolescents: A systematic review and Bayesian network meta-analysis**

**Table of Contents**

[*Supplementary file 1: Search strategy* 2](#_Toc179669220)

[*Supplementary file 2. Characteristics of included studies* 3](#_Toc179669221)

[*Supplementary file 3. Risk of bias in the included studies* 5](#_Toc179669222)

[*Supplementary file 4. Methods* 6](#_Toc179669223)

[S4.1 Eligibility Criteria 6](#_Toc179669224)

[S4.2 Data extraction and coding management 7](#_Toc179669225)

[S4.3 Statistical Analysis 7](#_Toc179669226)

[*Supplementary file 5. Pairwise analysis* 8](#_Toc179669227)

[S5.1 Details of pairwise meta-analyses 8](#_Toc179669228)

[S5.2 Comparison-adjusted funnel plot of all studies 9](#_Toc179669229)

[*Supplementary file 6. Network meta-analysis* 9](#_Toc179669230)

[S6.1 network diagrams 9](#_Toc179669231)

[S6.2 Table S3. Assessment of inconsistency within comparisons 10](#_Toc179669232)

[S6.3 Dot splitting method to explore inconsistency 11](#_Toc179669233)

[S6.4 Sensitivity analysis 11](#_Toc179669234)

[S6.5 GRADE Assessment 12](#_Toc179669235)

[*Supplementary file 7. Dose network meta-analysis* 13](#_Toc179669236)

[S7.1 Table S5. Dataset 13](#_Toc179669237)

[S7.2 Key assumptions of Network Meta-Analysis 16](#_Toc179669238)

[S7.3 Models’ selection 18](#_Toc179669239)

[S7.4 Dose-response relationships 21](#_Toc179669240)

[S7.5 Ranking of effectiveness of interventions 21](#_Toc179669241)

[*References* 23](#_Toc179669242)

*Supplementary file 8.* [*PRISMA NMA Checklist of Items to Include When Reporting A Systematic Review Involving a Network Meta-analysis* 26](#_Toc179669244)

[Supplementary file 1: Search strategy](#_heading=h.17dp8vu)

| Results | Search strategy | Database name |
| --- | --- | --- |
| 4328 | ("youth"[Title/Abstract] OR "youths"[Title/Abstract] OR "adolescent"[Title/Abstract] OR "adolescents"[Title/Abstract] OR "teenager"[Title/Abstract] OR "teenagers"[Title/Abstract] OR "child"[Title/Abstract] OR "children"[Title/Abstract] OR "student"[Title/Abstract] OR "boy"[Title/Abstract] OR "girl"[Title/Abstract]) AND ("physical activity"[Title/Abstract] OR "physical exercise"[Title/Abstract] OR "sport"[Title/Abstract] OR "motor"[Title/Abstract] OR "athletic sports"[Title/Abstract] OR "aerobic exercise"[Title/Abstract] OR "aerobic training"[Title/Abstract] OR "strength training"[Title/Abstract] OR "muscle-strengthening exercise"[Title/Abstract]OR "physical education"[Title/Abstract] OR "fitness game"[Title/Abstract]) AND ("depressive"[Title/Abstract] OR "depression"[Title/Abstract] OR "depressed"[Title/Abstract] OR "despair"[Title/Abstract] OR "dysphoria"[Title/Abstract] OR "melancholia"[Title/Abstract] OR "despondency"[Title/Abstract] OR "mental health"[Title/Abstract] OR "emotional depression"[Title/Abstract] OR "depressive symptom"[Title/Abstract]) | PubMed |
| 9552 | ((AB=(youth OR youths OR adolescent OR adolescents OR teenager OR teenagers OR child OR children OR student OR students OR boy OR girl)) AND AB=(“physical activity” OR “physical exercise” OR sport OR motor OR “athletic sports” OR “aerobic exercise” OR “aerobic training” OR “strength training” OR “muscle-strengthening exercise” OR “physical education” OR “fitness game”)) AND TS=(depressive OR depression OR depressed OR despair OR dysphoria OR melancholia OR despondency OR “‘mental health” OR “emotional depression” OR “depressive symptom”) | Web of Science |
| 1833 | AB ( youth OR youths OR adolescent OR adolescents OR teenager OR teenagers OR child OR children OR student OR students OR boy OR girl ) AND AB ( “physical activity” OR “physical exercise” OR sport OR motor OR “athletic sports” OR “aerobic exercise” OR “aerobic training” OR “strength training” OR “muscle-strengthening exercise” OR “physical education” OR “fitness game” ) AND AB ( depression OR depressive OR depressed OR melancholia OR dysphoria OR despair OR despondency OR “‘mental health” OR “emotional depression” OR “depressive symptom” ) | PsycINFO |
| 6715 | ( TITLE-ABS ( youth OR adolescent OR teenager OR child OR children OR student OR boy OR girl ) AND TITLE-ABS ( "physical activity" OR "physical exercise" OR sport OR motor OR "athletic sports" OR "aerobic exercise" OR "aerobic training" OR "strength training" OR "muscle-strengthening exercise" OR "physical education" OR "fitness game" ) AND TITLE-ABS ( depressive OR depression OR depressed OR despair OR dysphoria OR melancholia OR despondency OR "'mental health" OR "emotional depression" OR "depressive symptom" ) ) | Scopus |

Supplementary file 2. Characteristics of included studies

Table S1. Characteristics of included studies

| Author | Sample size(n) | Sample M_age_ (SD) | Intervention parameter | Intervention group condition | Outcome |
| --- | --- | --- | --- | --- | --- |
| Annesi, 2005 | 90 | 10.5±0.9 | Mixed training | 12 weeks  3 days per week  45 min per session | POMS-SF |
| Bonhauser et al., 2005 | 198 | 15 | Mixed training | 24 weeks  3 days per week  90 min per session | HADS |
| Butzer et al., 2017 | 201 | 12.64±0.33 | Mind-body exercise | 24 weeks  2 days per week  35 min per session | BRUMS |
| Carter et al., 2015 | 61 | 15.8±0.6 | Mixed training | 8 weeks  3 days per week  8-10 min per session | CDI-2 |
| Costigan et al., 2016 | 64 | 16.8±0.6 | G1: Aerobic exercise  G2: Mixed training | 6 weeks  2 days per week  30 min per session | K10 |
| Crews et al., 2004 | 66 | NA | Mixed training | 6 weeks  3 days per week  20 min per session | BDI |
| Daley et al., 2006 | 53 | 11-16 | Aerobic exercise | 8 weeks  3 days per week  40 min per session | CDI |
| Goldfield et al., 2018 | 224 | 15.6±1.4 | G1: Aerobic exercise  G2: Mixed training | 22 weeks  4 days per week  25 min per session | BRUMS |
| Hughes et al., 2013 | 26 | 15-18 | Aerobic exercise | 20 weeks  3 days per week  90 min per session | CDRS-R |
| Jeong et al., 2005 | 40 | 16 | Aerobic exercise | 12 weeks  3 days per week  45 min per session | SCL-90-R |
| Khalsa et al., 2012 | 121 | 16.8±0.6 | Mind-body exercise | 11 weeks  2-3 days per week  45 min per session | POMS-SF |
| Lin et al., 2020 | 39 | 12-14 | Aerobic exercise | 12 weeks  4 days per week  30 min per session | BPSS-R-Pt |
| MacMahon and Gross, 1988 | 69 | 14-18 | Mixed training | 12 weeks  3 days per week  40 min per session | BDI |
| Mohammadi and Abhar, 2011 | 80 | NA | Group training | 8 weeks  3 days per week  75 min per session | BDI |
| Nabkasorn et al., 2006 | 49 | NA | Aerobic exercise | 10 weeks  2 days per week  30 min per session | CES-D |
| Olive et al., 2019 | 406 | 8±0.6 | Mind-body exercise | 208 weeks  2 days per week  30 min per session | CSQ |
| Petty et al., 2009 | 201 | 7-11 | Mixed training | 13 weeks  7 days per week  40 min per session | RCDS |
| Romero-Pérez et al.,2020 | 105 | 17.5±2.24 | Mixed training | 6 weeks  3 days per week | CDS |
| Roth and Holmes, 1987 | 55 | 11.99±0.33 | G1: Aerobic exercise  G2: Mind-body exercise | 20 weeks  1 days per week  60 min per session | BDI |
| Shachar et al., 2016 | 649 | grades 3-6 | Group training | 24 weeks  120-180min per week | PANAS |
| Silva et al., 2020 | 33 | 11-14 | Aerobic exercise | 8 weeks  2 days per week  30 min per session | CDI |
| Talakoub et al., 2012 | 175 | 17.43±2.09 | Aerobic exercise | 6 weeks  3 days per week  60 min per session | SCL-90-R |
| Weintraub et al., 2008 | 21 | 9.50±0.58 | Group training | 24 weeks  3 days per week  75 min per session | CDI |
| Williams et al., 2019 | 175 | 9.7±0.9 | Mixed exercise | 56 weeks  7 days per week  40 min per session | CDI |
| Yu et al., 2020 | 171 | 9.8±0.7 | Mixed training | 32 weeks  5 days per week  40 min per session | SASC |
| Zhang et al., 2021 | 135 | 14.3±1.8 | Mixed training | 16 weeks  3 days per week  30 min per session | HAMD |

Supplementary file 3. Risk of bias in the included studies


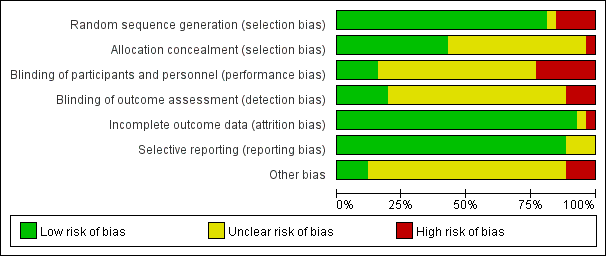


**Figure S1**. Risk of bias graph


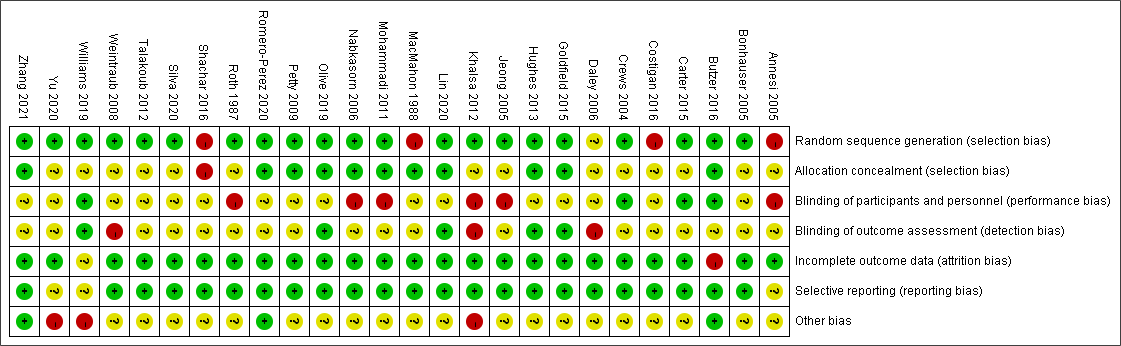


**Figure S2**. Risk of bias summary

Supplementary file 4. Methods

S4.1 Eligibility Criteria

The inclusion criteria of the literature followed the PICOS (Participants/Interventions/Comparisons/Outcomes/Study Design) principle. (1) Participants (P): Children and adolescents (under 18 years old) diagnosed with depression or mild depression/anxiety. (2) Intervention (I): Exercise is defined as planned, purposeful and repeatable physical activity (“WHO Guidelines Approved by the Guidelines Review Committee”, 2010). This study divided the exercises involved into four categories: aerobic exercise (mainly including running, dancing, cycling and other sports to improve cardiopulmonary health), group training (mainly including football, basketball, volleyball and other sports to cultivate team spirit), mixed training (training that integrates multiple sports), and mind-body exercise (mainly including yoga, martial arts and other sports to cultivate mind-body coordination and autonomy). (3) Comparison (C): Includes a control group without exercise intervention. For example, maintaining daily life and routine care. (4) Use a validated mature scale to investigate the effect of exercise on children and adolescents with depression. The results include the changes in depression scores before and after treatment in the exercise and non-intervention control groups (5) Study design (S): Randomized controlled trial.

Exclusion criteria include: (1) Adult patients with depression over 18 years old. (2) Exercise intervention combined with other interventions that may affect the outcome indicators. For example, psychological treatment or drug intervention. (3) Acute studies with an intervention duration of less than 6 weeks (3) Incomplete study data were not provided. (4) Conference articles, abstracts, and reviews that have not been peer-reviewed. (5) Cross-over design studies.

S4.2 Data extraction and coding management

We defined intensity per unit of exercise and calculated the formula as follows: intensity of a specific exercise type (Metabolic Equivalent of Task, MET) × time of a single exercise session × frequency of exercise per week. The result of the calculation is expressed in METs-min per week, which is the unit exercise intensity. The exercise specific intensities are referenced from the 2011 Compendium of Physical Activities (Ainsworth et al., 2011), which contains 821 activity-specific codes covering nearly all types of exercise. Exercise frequency was expressed as the total number of exercise interventions performed per week (including the number of exercises performed multiple times daily). Moreover, only the duration of the main intervention was extracted for exercise duration, excluding the warm-up and relaxation components. If the study reported a time range, we took the mean of the time range, and if the study did not report an intervention time, the study was excluded. Finally, to facilitate network analysis connectivity, weekly METs-min were approximated for each exercise intervention and categorized into specific categories: 0 (CON), 300, 600, 900, and 1200 MET-min per week. This process was necessary for the dosage NMA (Higgins et al., 2012).

S4.3 Statistical Analysis

***Pairwise meta-analysis***

The pairwise meta-analysis was conducted using a comprehensive meta-analysis. Separate pooled analyses were performed for each exercise modality group (AE, GT, MT, MBE) to determine the standardized mean difference (SMD) in depression scores between the exercise and control groups. Each exercise modality was then further dichotomized according to the classification of baseline depression and analyzed separately. Statistical heterogeneity was always performed concurrently with the pooled analyses and reported as the I² statistic. For I² statistic, when more than 50% corresponds to high heterogeneity (Higgins and Thompson, 2002). The presence of publication bias in the study was determined by examining the asymmetry of the corrected funnel plot and Egger’s test. (Lin et al., 2018). The results of the pooled analysis were considered significant with a p value of <0.05. All analyses were done in R language (version 4.3.3, package “meta”).

***Network Meta-analyses***

This study first drew a network diagram to preliminarily summarize the included studies and conducted a random-effects frequentist network meta-analysis (Harrer et al., 2021). We used standardized mean differences (SMDs) and 95% confidence intervals (CI) to estimate changes in depressive symptoms. In addition, we prepared forest plots and league tables to show the magnitude of the effect of exercise interventions on depression. The forest plots also ranked the treatment effects of different exercise methods on depression according to the P score (Rücker and Schwarzer, 2015). The P score was obtained by weighting the precision of direct and indirect comparisons in the network and then calculating the average of the probability that each intervention method was superior to all other intervention methods (Chaimani et al., 2013). The P score ranged from 0 to 1, and the higher the score, the better the improvement of depression by the exercise method (Efthimiou et al., 2016). Regarding heterogeneity, this study used τ² and I² statistics to assess network heterogeneity. We used prediction intervals in all forest plots, node splitting methods to assess network inconsistency, and “design-by treatment” models to assess network consistency (Dias et al., 2010; Higgins et al., 2012). Finally, this study conducted sensitivity analyses to ensure that the results of the network meta-analysis were robust and reliable. The network meta-analysis was conducted using the “netmeta” package in R software.

***Dose-response Network Meta-analyses***

To investigate the dose-response relationship between exercise and depression in children and adolescents, we conducted a dose-response analysis using a random-effects Bayesian model-based network meta-analysis (MBNMA)(Mawdsley et al., 2016) There was no evidence that the analysis violated the key assumptions of the MBNMA (network transitivity (Higgins et al., 2012), data consistency (Wheeler et al., 2010), and network connectivity (Ter Veer et al., 2019) (Appendix S7.2). We use some recommended dose-response functions to model (Emax, Restricted cubic splines, Quadratic function, Non-parametric) (Pedder et al., 2019) and compare the different functions' fit indices (Deviation Information Criterion (DIC), standard deviation between studies, parameters in the model, and residual deviance) (Evans, 2019). We resorted to Hedges’g standardized mean difference (SMD) coupled with 95% credible intervals (Crl) to represent the effect sizes and their credibility. The quadratic function model showed the best fit in all cases and was used to assess the nonlinear dose-response relationship (Appendix S7.3.2). Quadratic random effects models converge well in nonlinear relationships, and quadratic models usually reflect the phenomenon better than linear models in time- and dose-dependent models (Shim and Lee, 2019). The Beta coefficient in the model was used to estimate the dose of exercise that had a significant effect on depression and to rank the type of exercise in terms of efficacy based on the likelihood of triggering a change (Mawdsley et al., 2016). The “MBNMAdose” package in R language (version 4.3.3) was used to execute dose-response analysis and dose network meta-regression.

Supplementary file 5. Pairwise analysis

S5.1 Details of pairwise meta-analyses

**Table S2.** Details of pairwise meta-analyses

| Comparison | k | SMD | 95%CI |
| --- | --- | --- | --- |
| AE vs MT | 2 | -0.42 | (-1.62, 0.79) |
| AE vs MBE | 1 | -1.27 | (-4.86, 2.32) |
| AE vs CON | 10 | -0.60 | **(-1.10, -0.10)** |
| GT vs CON | 3 | -1.58 | **(-2.54, -0.62)** |
| MT vs CON | 12 | -0.32 | (-0.77, 0.14) |
| MBE vs CON | 4 | 0.04 | (-0.69, 0.77) |
| Comparison | k | SMD | 95%CI |

***Note:*** Bolded numbers indicate the presence of significance. AE, Aerobic exercise; GT, Group training; MT, Mix training; MBE, Mind-body exercise.

S5.2 Comparison-adjusted funnel plot of all studies


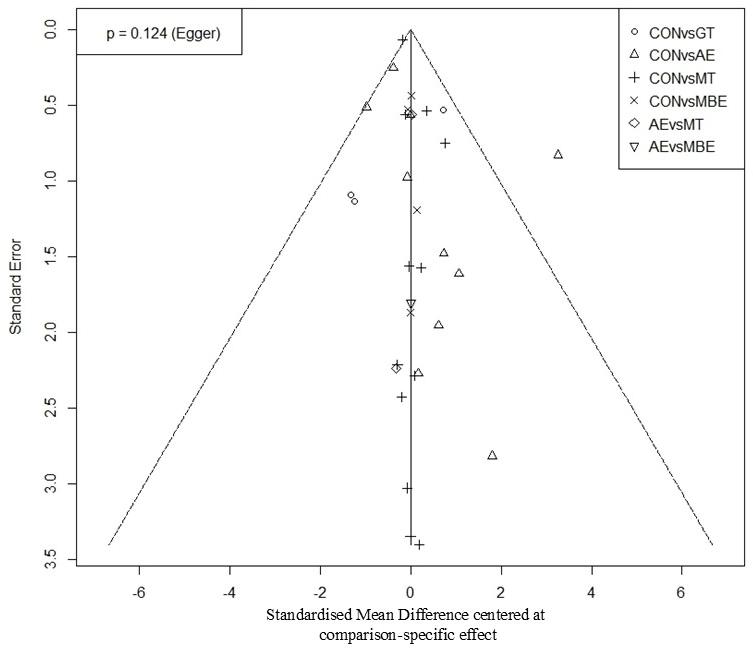


**Figure S3.** Comparison-adjusted funnel plot of all studies. AE, Aerobic exercise; GT, Group training; MT, Mix training; MBE, Mind-body exercise.

Supplementary file 6. Network meta- analysis

S6.1 network diagrams


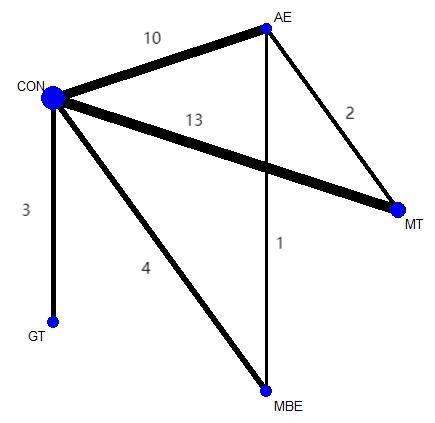


**Figure S4**. Comparative network diagram of all studies included in the network meta-analysis. Line width is proportional to the number of pairwise effect size estimates and node size is proportional to the number of participants.

S6.2 Table S3. Assessment of inconsistency within comparisons

| Comparison | k | Direct | Indirect | Difference | z | P-value |
| --- | --- | --- | --- | --- | --- | --- |
| AE vs GT | NA | NA | -0.96 | NA | NA | NA |
| AE vs MT | 2 | -0.42 | -0.27 | -0.15 | -0.20 | 0.839 |
| AE vs MBE | 1 | -1.27 | -0.64 | -0.63 | -0.37 | 0.707 |
| AE vs CON | 10 | -0.60 | -0.96 | 0.36 | 0.31 | 0.753 |
| GT vs MT | NA | NA | -1.27 | NA | NA | NA |
| GT vs MBE | NA | NA | -1.64 | NA | NA | NA |
| GT vs CON | 3 | -1.58 | NA | NA | NA | NA |
| MT vs MBE | NA | NA | -0.29 | NA | NA | NA |
| MT vs CON | 12 | -0.32 | -0.19 | -0.13 | -0.10 | 0.917 |
| MBE vs CON | 4 | 0.04 | 1.11 | -1.07 | -0.39 | 0.693 |

***Note:*** AE, Aerobic exercise; GT, Group training; MT, Mix training; MBE, Mind-body exercise.

S6.3 Dot splitting method to explore inconsistency


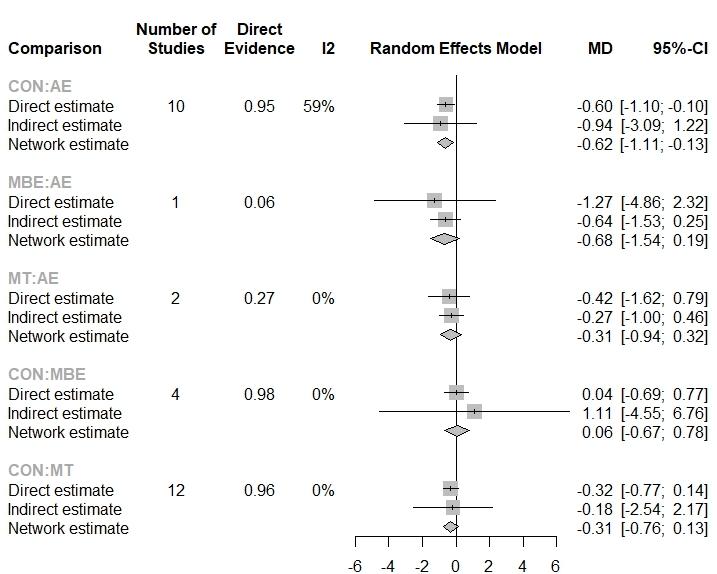


**Figure S5.** Dot splitting method to explore inconsistency. *I*^2^ represents the heterogeneity of pairwise comparisons and the red region represents the prediction interval. AE, Aerobic exercise; GT, Group training; MT, Mix training; MBE, Mind-body exercise

S6.4 Sensitivity analysis

**6.4.1 Forest plot for sensitivity analysis**


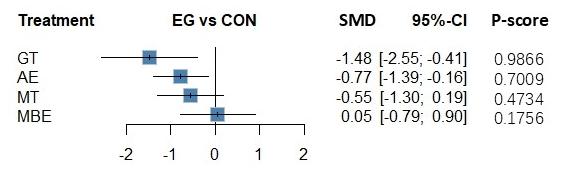


**Figure S6.** Forest plot of sensitivity analyses. AE, Aerobic exercise; GT, Group training; MT, Mix training; MBE, Mind-body exercise.

S6.5 GRADE Assessment

Table S4. Details of GRADE assessment for all pairwise comparisons within the depression network.

| Comparison | k | Direct SMD  [95% CI] | Quality of evidence | Indirect SMD  [95% CI] | Quality of evidence | Network SMD  [95% CI] | Quality of evidence | P-value |
| --- | --- | --- | --- | --- | --- | --- | --- | --- |
| AE vs GT | NA | NA |  | -0.96 (-2.03, 0.11) | Low*† | -0.96 (-2.03, 0.11) | Low*† | NA |
| AE vs MT | 2 | -0.42 (-1.62, 0.79) | Very low*‡ | -0.27 (-1.01, 0.38) | Very low*‡ | -0.31 (-0.94, 0.32) | Very low*‡ | 0.839 |
| AE vs MBE | 1 | -1.27 (-4.86, 2.32) | Very low*‡ | -0.64 (-1.53, 0.25) | Low*† | -0.60(-1.47, 0.27) | Very low*‡ | 0.707 |
| AE vs CON | 10 | -0.60 (-1.10, -0.10) | Low*† | -0.96 (-3.12, 1.20) | Moderate* | -0.62 (-1.11, -0.13) | Moderate* | 0.753 |
| GT vs MT | NA | NA |  | -1.27 (-2.32, -0.21) | Low*† | -1.27 (-2.32, -0.21) | Low*† | NA |
| GT vs MBE | NA | NA |  | -1.64 (-2.84, -0.44) | Low*† | -1.64 (-2.84, -0.44) | Low*† | NA |
| GT vs CON | 3 | -1.58 (-2.54, -0.62) | Low*† | NA |  | -1.58 (-2.54, -0.62) | Low*† | NA |
| MT vs MBE | NA | NA |  | -0.37 (-1.22, 0.48) | Very low*‡ | -0.37 (-1.22, 0.48) | Very low*‡ | NA |
| MT vs CON | 12 | -0.32 (-0.77, 0.14) | Moderate* | -0.19 (-2.55, 2.17) | Low*† | -0.31 (-0.78, -0.13) | Moderate* | 0.917 |
| MBE vs CON | 4 | 0.04 (-0.69, 0.77) | Very low*‡ | 1.11 (-4.55, 6.76) | Low*† | 0.06 (-0.67, 0.78) | Low*† | 0.693 |

k = Number of studies providing direct evidence, Network (SMD) = estimated treatment effect (SMD) in network meta-analysis, direct (SMD) = estimated treatment effect (SMD) derived from direct evidence, indirect (SMD) = estimated treatment effect (SMD) derived from indirect evidence, p = p-value of test for disagreement (difference between direct and indirect evidence)

Reasons for downgrading: †Imprecision (1 downgrade), ‡Severe imprecision (2 downgrades), *risk of bias (1 downgrade)

The p-value indicated that the findings between direct and indirect comparisons was not statistically significant and therefore no comparisons have been downgraded based on inconsistency.

Supplementary file 7. Dose network meta- analysis

S7.1 Table S5. Dataset

This Appendix shows the datasets (i.e., at global exercise and agent levels) used in this study. The studyID indicates the name of the author and the year of publication. The y indicates the mean change from the baseline of the evaluation tool. The SE indicates the standard error of the mean. The agent indicates the agent level. The exact_dose parameter indicates the exact estimated METs per week that participants accumulated in the study. The dose indicates the group of doses by approximation. The residual_dose indicates the difference between the exact dose and the dose allocated by approximation. The frequency is the number of days those participants were involved in physical activity. The min_day indicates how many minutes a session lasted. The N indicates the number of participants.

| Study | Agent | Exact dose | Dose | Residual dose | Frequency | Min/day | y | SE | N |
| --- | --- | --- | --- | --- | --- | --- | --- | --- | --- |
| Annesi 2005 | MT | 810 | 900 | 90 | 3 | 45 | -1.9 | 0.5 | 49 |
| Annesi 2005 | CON | 0 | 0 | 0 | 0 | 0 | -0.1 | 0.6 | 41 |
| Bonhauser 2005 | MT | 1890 | 1200 | 690 | 3 | 90 | -0.05 | 0.1 | 98 |
| Bonhauser 2005 | CON | 0 | 0 | 0 | 0 | 0 | 0.02 | 0.1 | 100 |
| Butzer 2016 | MBE | 280 | 300 | 20 | 2 | 35 | -3.49 | 0.4 | 110 |
| Butzer 2016 | CON | 0 | 0 | 0 | 0 | 0 | -3.49 | 0.4 | 91 |
| Carter 2015 | MT | 630 | 600 | 30 | 3 | 8-10 | -10.63 | 1.8 | 32 |
| Carter 2015 | CON | 0 | 0 | 0 | 0 | 0 | -5.82 | 2.0 | 29 |
| Costigan 2016 | AE | 202.5 | 300 | 97.5 | 2 | 30 | -0.43 | 1.6 | 21 |
| Costigan 2016 | MT | 189 | 300 | 111 | 2 | 30 | -0.13 | 1.5 | 22 |
| Costigan 2016 | CON | 0 | 0 | 0 | 0 | 0 | 0.05 | 1.6 | 21 |
| Crews 2004 | MT | 360 | 300 | 60 | 3 | 20 | -2.1 | 1.6 | 34 |
| Crews 2004 | CON | 0 | 0 | 0 | 0 | 0 | -1.2 | 1.8 | 32 |
| Daley 2006 | AE | 540 | 600 | 60 | 3 | 40 | -3.11 | 1.1 | 28 |
| Daley 2006 | CON | 0 | 0 | 0 | 0 | 0 | -1.88 | 1.2 | 25 |
| Goldfield 2015 | AE | 1080 | 900 | 180 | 4 | 25 | -0.7 | 0.4 | 78 |
| Goldfield 2015 | MT | 1440 | 1200 | 240 | 4 | 25 | -0.7 | 0.4 | 71 |
| Goldfield 2015 | CON | 0 | 0 | 0 | 0 | 0 | 0 | 0.4 | 75 |
| Hughes 2013 | AE | 720 | 600 | 120 | 3 | 90 | -3 | 0.2 | 14 |
| Hughes 2013 | CON | 0 | 0 | 0 | 0 | 0 | -2.6 | 0.2 | 12 |
| Jeong 2005 | AE | 675 | 600 | 75 | 3 | 45 | -5.4 | 2.5 | 20 |
| Jeong 2005 | CON | 0 | 0 | 0 | 0 | 0 | 2.5 | 1.3 | 20 |
| Khalsa 2012 | MBE | 297 | 300 | 3 | 2-3 | 45 | -0.34 | 0.7 | 74 |
| Khalsa 2012 | CON | 0 | 0 | 0 | 0 | 0 | 0.33 | 1.0 | 47 |
| Lin 2020 | AE | 660 | 600 | 60 | 4 | 30 | -2.3 | 1.0 | 20 |
| Lin 2020 | CON | 0 | 0 | 0 | 0 | 0 | -0.7 | 1.0 | 19 |
| MacMahon 1988 | MT | 1080 | 900 | 180 | 3 | 40 | -20.38 | 1.6 | 32 |
| MacMahon 1988 | CON | 0 | 0 | 0 | 0 | 0 | -20.05 | 1.6 | 37 |
| Mohammadi 2011 | GT | 1800 | 1200 | 600 | 3 | 75 | -7.95 | 0.8 | 40 |
| Mohammadi 2011 | CON | 0 | 0 | 0 | 0 | 0 | -5 | 1.0 | 40 |
| Nabkasorn 2006 | AE | 1250 | 1200 | 50 | 2 | 30 | -3.1 | 0.4 | 21 |
| Nabkasorn 2006 | CON | 0 | 0 | 0 | 0 | 0 | -3.8 | 0.3 | 28 |
| Olive 2019 | MBE | 520 | 600 | 80 | 2 | 30 | -0.54 | 0.3 | 217 |
| Olive 2019 | CON | 0 | 0 | 0 | 0 | 0 | -0.68 | 0.3 | 189 |
| Petty 2009 | MT | 850 | 900 | 50 | 7 | 40 | -3.8 | 1.2 | 63 |
| Petty 2009 | MT | 1700 | 1200 | 500 | 7 | 40 | -2.6 | 0.8 | 70 |
| Petty 2009 | CON | 0 | 0 | 0 | 0 | 0 | -1.4 | 1.4 | 68 |
| Romero-Pérez 2020 | MT | 600 | 600 | 0 | 3 | 40 | -1 | 0.3 | 54 |
| Romero-Pérez 2020 | CON | 0 | 0 | 0 | 0 | 0 | 0 | 1.9 | 51 |
| Roth 1987 | AE | 540 | 600 | 60 | 1 | 60 | -3.7 | 1.3 | 18 |
| Roth 1987 | MBE | 270 | 300 | 30 | 1 | 60 | -1.5 | 1.2 | 19 |
| Roth 1987 | CON | 0 | 0 | 0 | 0 | 0 | -2 | 1.4 | 18 |
| Shachar 2016 | GT | 1012.5 | 900 | 112.5 | 1 | 120-180 | -25.4 | 0.3 | 330 |
| Shachar 2016 | CON | 0 | 0 | 0 | 0 | 0 | -20.5 | 0.4 | 319 |
| Silva 2020 | AE | 540 | 600 | 60 | 2 | 30 | -6.9 | 0.5 | 18 |
| Silva 2020 | CON | 0 | 0 | 0 | 0 | 0 | 1.1 | 0.7 | 15 |
| Talakoub 2012 | AE | 1080 | 900 | 180 | 3 | 60 | -1.3 | 0.6 | 90 |
| Talakoub 2012 | CON | 0 | 0 | 0 | 0 | 0 | -1.3 | 0.7 | 85 |
| Weintraub 2008 | GT | 2025 | 1200 | 825 | 3 | 75 | -1.62 | 0.7 | 9 |
| Weintraub 2008 | CON | 0 | 0 | 0 | 0 | 0 | -1.16 | 0.9 | 12 |
| Williams 2019 | MT | 1680 | 1200 | 480 | 7 | 40 | -1.3 | 0.1 | 90 |
| Williams 2019 | CON | 0 | 0 | 0 | 0 | 0 | -1.3 | 0.7 | 85 |
| Yu 2020 | MT | 1300 | 1200 | 100 | 5 | 40 | -11 | 2.4 | 99 |
| Yu 2020 | CON | 0 | 0 | 0 | 0 | 0 | -3 | 2.3 | 72 |
| Zhang 2021 | MT | 405 | 300 | 105 | 3 | 30 | -27.11 | 0.4 | 66 |
| Zhang 2021 | CON | 0 | 0 | 0 | 0 | 0 | -23.17 | 0.4 | 69 |

S7.2 Key assumptions of Network Meta-Analysis

The following are the three key assumptions of the network meta-analysis.

**S7.2.1 Connectivity**

Connectivity is a key assumption in NMA that, if considered insufficient (i.e., due to lack of direct comparisons), may lead to low statistical power and misleading results. Our study assessed network connectivity at the motor and dose levels and did not find any evidence of network unconnectedness (**Figure S7** and **Figure S8**).


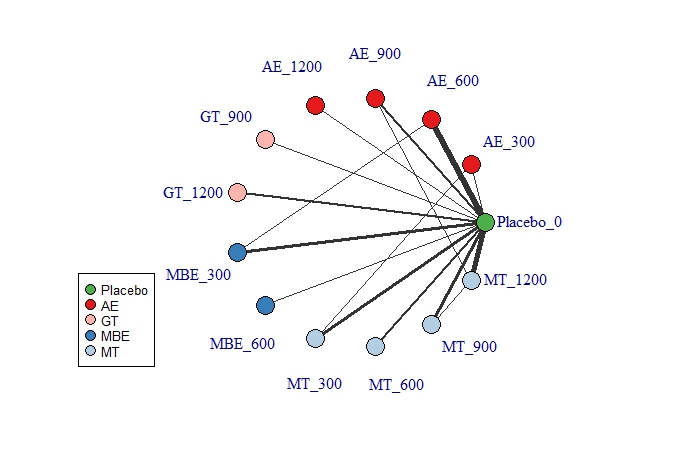


**Figure S7.** Treatment-level network. The first value indicates the specific intervention and the second one is the corresponding dose of that intervention. AE, Aerobic exercise; GT, Group training; MT, Mix training; MBE, Mind-body exercise; Placebo, Control.


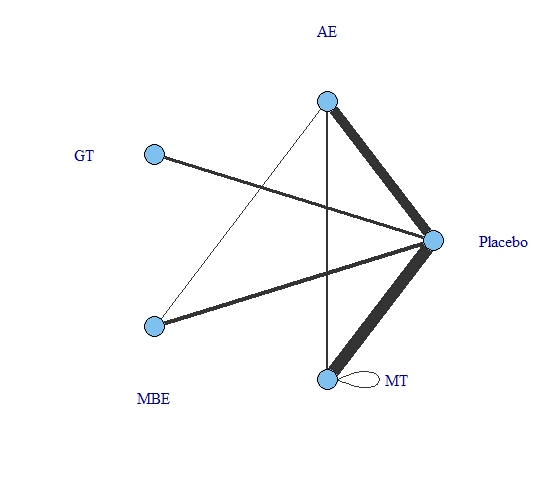


**Figure S8.** Agent-level network. AE, Aerobic exercise; GT, Group training; MT, Mix training; MBE, Mind-body exercise; Placebo, Control.

**S7.2.2 Consistency**

We performed a consistency analysis of the data by comparing the consistency of the network (i.e., network effect size) with the unrelated mean effects (UME) model (i.e., pairwise effect sizes). In practice, we checked whether the bias, the number of estimated parameters in the network, and the Deviance Information Criterion (DIC) metric were similar for both models, indicating a good fit (Wheeler et al., 2010). The comparison of these parameters showed a good agreement between the different models (Table S6).

**Table S6.** Consistent and UME models fit comparison

| Model | pD | Deviance | Residual deviance | DIC |
| --- | --- | --- | --- | --- |
| Consistent | 37.8 | 116.1 | 47.5 | 153.9 |
| UME | 38 | 115.3 | 46.7 | 153.3 |

***Note***: pD: Number of estimated parameters; DIC: Deviance Informative Criterion; SD: Standard

Deviation; UME: Unrelated Mean Effects. Scientific literature indicated that the main indicator to

assess the model fit is the DIC. As lower DIC, better fit.

**S7.2.3 Transitivity**

NMAs are based on the assumption of indirect/mixed comparisons, which implies that estimates of treatment effects from direct and indirect evidence are consistent but with the usual variation of meta-analyses under a random effects model (White et al., 2012). This assumption is equivalent to heterogeneity in a "standard" meta-analysis (Cipriani et al., 2013). Following a previous proposal (Salanti et al., 2014), anomalies were assessed at a deeper network level (i.e., at the treatment level). We assessed the span by the MBNMA node splitting method. This method splits the contribution of a specific treatment contrast into direct and indirect evidence and compares them (Van Valkenhoef et al., 2016) . Similar effects indicate good span. **Table S7** presents the results for transitivity in this meta-analysis.

**Table S7.** Node-splitting analysis of inconsistency

| Comparison | P-value | Median | 95%CrI | |
| --- | --- | --- | --- | --- |
| AE_300 vs Placebo_0 | 0.136 |  |  |  |
| -> direct |  | -0.589 | -5.474 | 4.084 |
| -> indirect |  | -0.231 | -0.589 | 0.036 |
| -> MBNMA |  | -0.240 | -0.553 | 0.040 |
| AE_600 vs Placebo_0 | 0.154 |  |  |  |
| -> direct |  | -1.393 | -2.661 | -0.326 |
| -> indirect |  | -0.130 | -0.833 | 0.518 |
| -> MBNMA |  | -0.480 | -1.107 | 0.079 |
| AE_900 vs Placebo_0 | 0.797 |  |  |  |
| -> direct |  | -0.591 | -2.292 | 1.056 |
| -> indirect |  | -0.813 | -2.093 | 0.180 |
| -> MBNMA |  | -0.720 | -1.660 | 0.119 |
| AE_1200 vs Placebo_0 | 0.202 |  |  |  |
| -> direct |  | 0.354 | -1.545 | 2.270 |
| -> indirect |  | -1.668 | -3.290 | -0.321 |
| -> MBNMA |  | -0.961 | -2.213 | 0.158 |
| GT_900 vs Placebo_0 | 0.176 |  |  |  |
| -> direct |  | -2.309 | -4.028 | -0.481 |
| -> indirect |  | -0.210 | -1.576 | 1.172 |
| -> MBNMA |  | -1.047 | -2.120 | 0.174 |
| GT_1200 vs Placebo_0 | 0.179 |  |  |  |
| -> direct |  | -0.237 | -2.096 | 1.637 |
| -> indirect |  | -3.087 | -5.423 | -0.716 |
| -> MBNMA |  | -1.396 | -2.827 | 0.232 |
| MBE_300 vs Placebo_0 | 0.789 |  |  |  |
| -> direct |  | 0.120 | -1.361 | 1.608 |
| -> indirect |  | 0.017 | -1.041 | 0.991 |
| -> MBNMA |  | 0.044 | -0.757 | 0.836 |
| MBE_600 vs Placebo_0 | 0.783 |  |  |  |
| -> direct |  | -0.009 | -2.000 | 2.023 |
| -> indirect |  | 0.256 | -2.937 | 3.101 |
| -> MBNMA |  | 0.088 | -1.515 | 1.673 |
| MT_300 vs Placebo_0 | 0.178 |  |  |  |
| -> direct |  | -2.140 | -5.206 | 1.143 |
| -> indirect |  | -0.368 | -0.914 | 0.134 |
| -> MBNMA |  | -0.417 | -0.946 | 0.081 |
| MT_600 vs Placebo_0 | 0.272 |  |  |  |
| -> direct |  | -0.464 | -2.116 | 1.260 |
| -> indirect |  | -0.092 | -0.353 | 0.142 |
| -> MBNMA |  | -0.095 | -0.346 | 0.124 |
| MT_900 vs Placebo_0 | 0.461 |  |  |  |
| -> direct |  | -0.402 | -3.083 | 2.137 |
| -> indirect |  | -0.299 | -1.154 | 0.424 |
| -> MBNMA |  | -0.286 | -1.039 | 0.372 |
| MT_1200 vs Placebo_0 | 0.484 |  |  |  |
| -> direct |  | -0.324 | -1.368 | 0.596 |
| -> indirect |  | -0.917 | -3.615 | 2.107 |
| -> MBNMA |  | -0.381 | -1.386 | 0.496 |

S7.3 Models’ selection

**S7.3.1 Non-linear functions and models fit comparison**

A meta-analysis (i.e., a "split" NMA) of the different doses of physical activity as separate and unrelated treatments were performed. This step helps determine which function is more appropriate for the data and should be used in a model-based network meta-analysis (MBNMA) (Mawdsley et al., 2016) **Figure S9** and **Figure S10** show the different responses of each dose to overall and different types of exercise, respectively (Hedges' g).


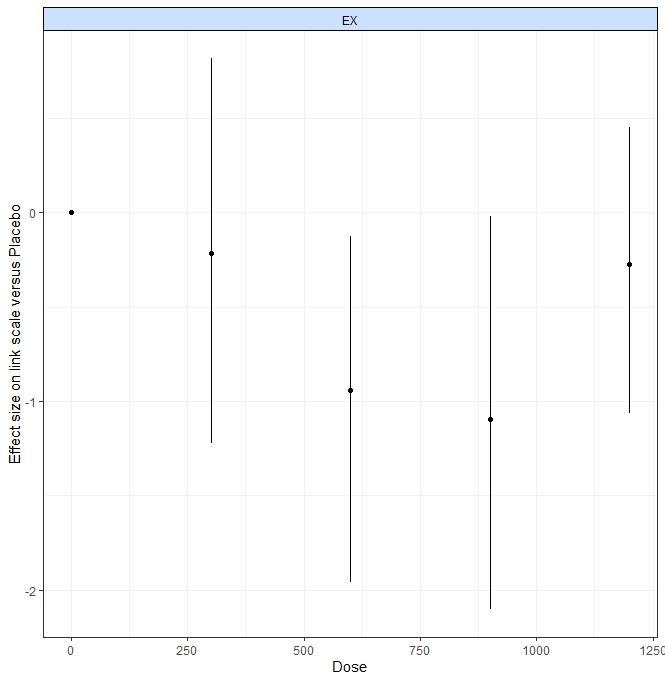


**Figure S9.** “Split” NMA of overall exercise


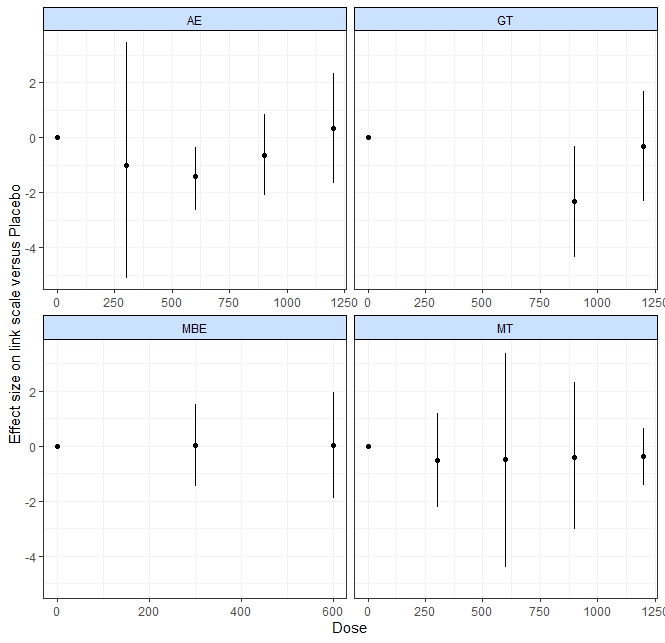


**Figure S10**. “Split” NMA of different exercise agents. AE, Aerobic exercise; GT, Group training; MT, Mix training; MBE, Mind-body exercise.

**S7.3.2 Models selection**

Quadratic model shows the best fit and were therefore used in subsequent analyses**（Table S8）**.

**Table S8** Models fit comparison

| Model | DIC | SD | Deviance | Residual deviance | pD |
| --- | --- | --- | --- | --- | --- |
| Emax (random treatment effects) | 154.1 | NA | 116.2 | 47.6 | 37.9 |
| Restricted cubic spline (common treatment effects; 3 knots) | 164.8 | NA | 126.7 | 58.2 | 38.1 |
| Restricted cubic spline (random treatment effects; 3 knots) | 158.8 | 0.762  (0.178, 1.5) | 115.2 | 46.6 | 43.6 |
| Exponential (common treatment effects) | 156.7 | NA | 126.9 | 58.3 | 29.9 |
| Quadratic (random treatment effects) | 151 | 0.677  (0.274,1.16) | 113.7 | 45.1 | 37.3 |

*Note.* DIC = Deviance Information Criterion; SD = Between-study Standard Deviation; pD: Number of estimated parameters; NA = Not Applicable. The SD is presented as the main value and (95% Credible Intervals).

In addition to the model fit index, a deviation plot showing the contribution of each data point to the residuals can also help to confirm the robustness of the model selection. The contribution of each data point to the posterior mean bias should be around 1, which indicates a good model fit (Dias et al., 2013). Deviation plots for overall **(Figure S11)** and treatment effects **(Figure S12)** confirm the robustness of our model selection (i.e., deviations <1.5 except for a few data points in the overall exercise and AE at 600 METs-min, which are all below a contribution of 1.5).


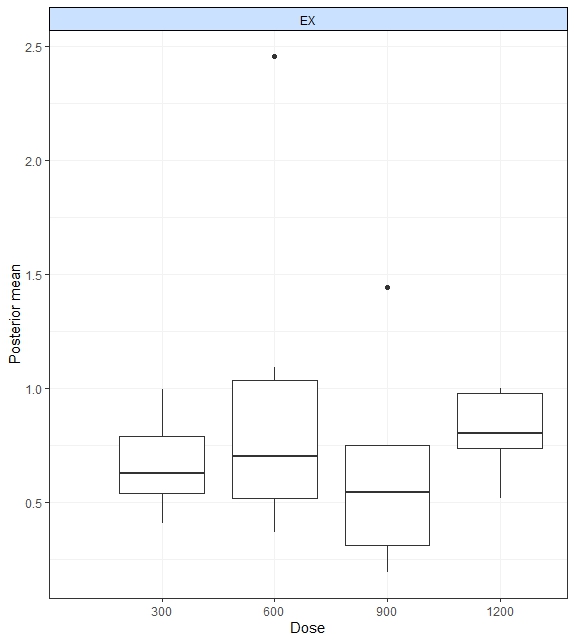


**Figure S11**. Deviance plot at overall exercise level.


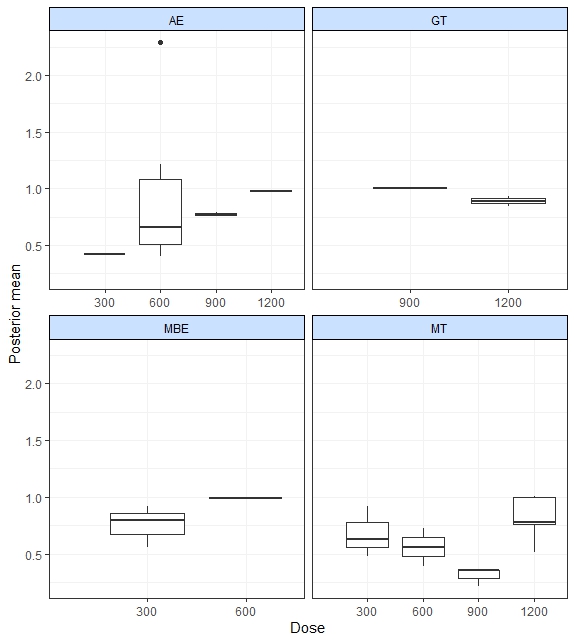


**Figure S12**. Deviance plots at treatment-level.

S7.4 Dose-response relationships


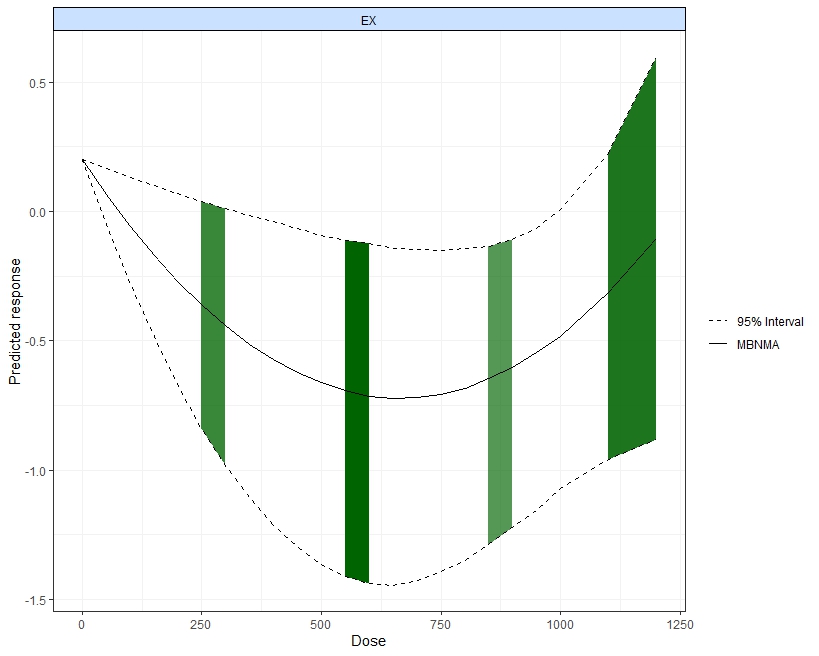


**Figure S13**. Exercise dose-response relationship at agent-level.


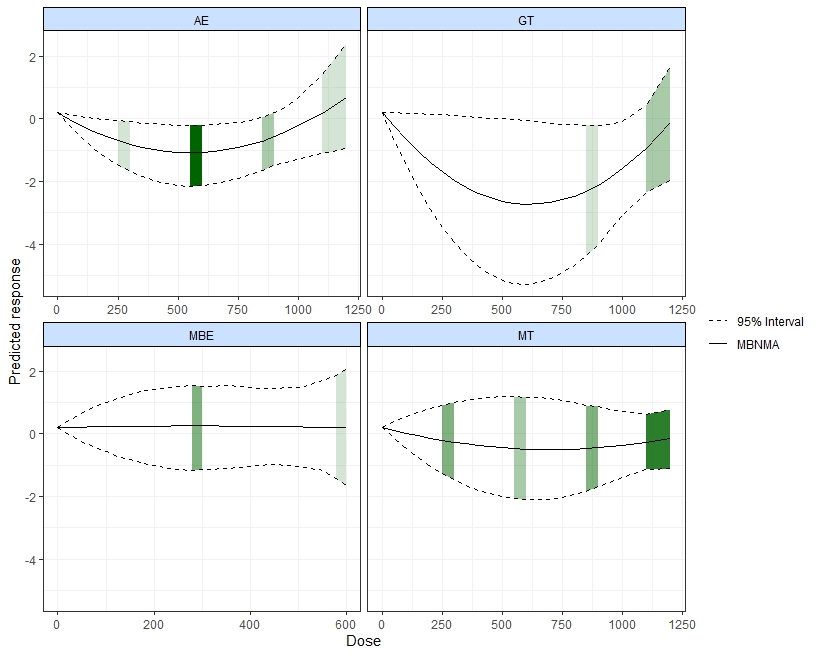


**Figure S14**. Number of original studies at different dose scenarios, the darker the color, the larger the amount of data.

S7.5 Ranking of effectiveness of interventions

**Figure S15** shows the probability of each intervention to be ranked from worst to best (estimated after up to 4000 iterations).


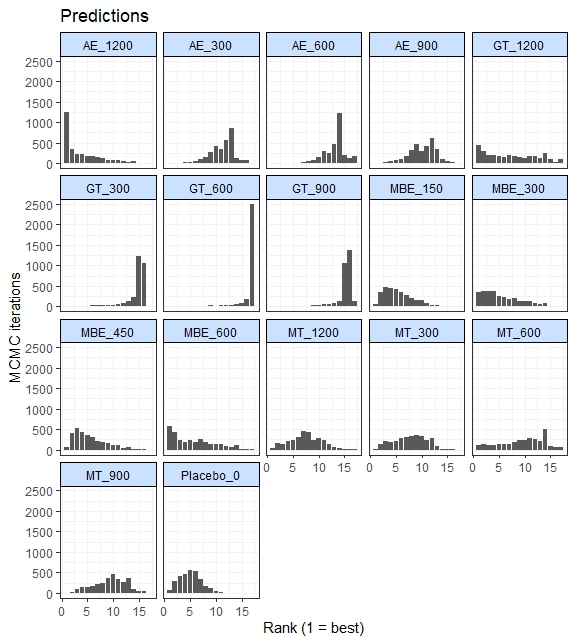


**Figure S15** Effectiveness ranking by exercise treatments. The number that follows the exercise intervention indicates the dose of exercise (METs-min/week).

References

Ainsworth, B.E., Haskell, W.L., Herrmann, S.D., Meckes, N., Bassett, D.R., Tudor-Locke, C., Greer, J.L., Vezina, J., Whitt-Glover, M.C., Leon, A.S., 2011. 2011 Compendium of Physical Activities: a second update of codes and MET values. Med Sci Sports Exerc 43, 1575–1581. https://doi.org/10.1249/MSS.0b013e31821ece12

Annesi, J.J., 2005. Correlations of depression and total mood disturbance with physical activity and self-concept in preadolescents enrolled in an after-school exercise program. Psychol Rep 96, 891–898. https://doi.org/10.2466/pr0.96.3c.891-898

Bonhauser, M., Fernandez, G., Püschel, K., Yañez, F., Montero, J., Thompson, B., Coronado, G., 2005. Improving physical fitness and emotional well-being in adolescents of low socioeconomic status in Chile: results of a school-based controlled trial. Health Promot Int 20, 113–122. https://doi.org/10.1093/heapro/dah603

Butzer, B., LoRusso, A., Shin, S.H., Khalsa, S.B.S., 2017. Evaluation of Yoga for Preventing Adolescent Substance Use Risk Factors in a Middle School Setting: A Preliminary Group-Randomized Controlled Trial. J Youth Adolesc 46, 603–632. https://doi.org/10.1007/s10964-016-0513-3

Carter, T., Guo, B., Turner, D., Morres, I., Khalil, E., Brighton, E., Armstrong, M., Callaghan, P., 2015. Preferred intensity exercise for adolescents receiving treatment for depression: a pragmatic randomised controlled trial. BMC Psychiatry 15, 247. https://doi.org/10.1186/s12888-015-0638-z

Chaimani, A., Higgins, J.P.T., Mavridis, D., Spyridonos, P., Salanti, G., 2013. Graphical tools for network meta-analysis in STATA. PLoS One 8, e76654. https://doi.org/10.1371/journal.pone.0076654

Cipriani, A., Higgins, J.P.T., Geddes, J.R., Salanti, G., 2013. Conceptual and technical challenges in network meta-analysis. Ann Intern Med 159, 130–137. https://doi.org/10.7326/0003-4819-159-2-201307160-00008

Costigan, S.A., Eather, N., Plotnikoff, R.C., Hillman, C.H., Lubans, D.R., 2016. High-Intensity Interval Training for Cognitive and Mental Health in Adolescents. Med Sci Sports Exerc 48, 1985–1993. https://doi.org/10.1249/MSS.0000000000000993

Crews, D.J., Lochbaum, M.R., Landers, D.M., 2004. Aerobic physical activity effects on psychological well-being in low-income Hispanic children. Percept Mot Skills 98, 319–324. https://doi.org/10.2466/pms.98.1.319-324

Daley, A.J., Copeland, R.J., Wright, N.P., Roalfe, A., Wales, J.K.H., 2006. Exercise therapy as a treatment for psychopathologic conditions in obese and morbidly obese adolescents: a randomized, controlled trial. Pediatrics 118, 2126–2134. https://doi.org/10.1542/peds.2006-1285

Dias, S., Sutton, A.J., Ades, A.E., Welton, N.J., 2013. Evidence Synthesis for Decision Making 2: A Generalized Linear Modeling Framework for Pairwise and Network Meta-analysis of Randomized Controlled Trials. Medical Decision Making 33, 607–617. https://doi.org/10.1177/0272989X12458724

Dias, S., Welton, N.J., Caldwell, D.M., Ades, A.E., 2010. Checking consistency in mixed treatment comparison meta-analysis. Stat Med 29, 932–944. https://doi.org/10.1002/sim.3767

Efthimiou, O., Debray, T.P.A., van Valkenhoef, G., Trelle, S., Panayidou, K., Moons, K.G.M., Reitsma, J.B., Shang, A., Salanti, G., Group, on behalf of G.M.R., 2016. GetReal in network meta-analysis: a review of the methodology. Research Synthesis Methods 7, 236–263. https://doi.org/10.1002/jrsm.1195

Evans, N.J., 2019. Assessing the practical differences between model selection methods in inferences about choice response time tasks. Psychon Bull Rev 26, 1070–1098. https://doi.org/10.3758/s13423-018-01563-9

Goldfield, G.S., Kenny, G.P., Prud’homme, D., Holcik, M., Alberga, A.S., Fahnestock, M., Cameron, J.D., Doucette, S., Hadjiyannakis, S., Tulloch, H., Tremblay, M.S., Walsh, J., Guerin, E., Gunnell, K.E., D’Angiulli, A., Sigal, R.J., 2018. Effects of aerobic training, resistance training, or both on brain-derived neurotrophic factor in adolescents with obesity: The hearty randomized controlled trial. Physiology & Behavior 191, 138–145. https://doi.org/10.1016/j.physbeh.2018.04.026

Harrer, M., Cuijpers, P., Furukawa, T., Ebert, D., 2021. Doing Meta-Analysis with R: A Hands-On Guide. Chapman and Hall/CRC, New York. https://doi.org/10.1201/9781003107347

Higgins, J.P.T., Jackson, D., Barrett, J.K., Lu, G., Ades, A.E., White, I.R., 2012. Consistency and inconsistency in network meta-analysis: concepts and models for multi-arm studies. Res Synth Methods 3, 98–110. https://doi.org/10.1002/jrsm.1044

Higgins, J.P.T., Thompson, S.G., 2002. Quantifying heterogeneity in a meta-analysis. Stat Med 21, 1539–1558. https://doi.org/10.1002/sim.1186

Hughes, C.W., Barnes, S., Barnes, C., Defina, L.F., Nakonezny, P., Emslie, G.J., 2013. Depressed Adolescents Treated with Exercise (DATE): A pilot randomized controlled trial to test feasibility and establish preliminary effect sizes. Ment Health Phys Act 6. https://doi.org/10.1016/j.mhpa.2013.06.006

Jeong, Y.-J., Hong, S.-C., Lee, M.S., Park, M.-C., Kim, Y.-K., Suh, C.-M., 2005. Dance movement therapy imporves emotional responses and modulates neurohormones in adolescents with mild depression. International Journal of Neuroscience 115, 1711–1720. https://doi.org/10.1080/00207450590958574

Khalsa, S.B.S., Hickey-Schultz, L., Cohen, D., Steiner, N., Cope, S., 2012. Evaluation of the Mental Health Benefits of Yoga in a Secondary School: A Preliminary Randomized Controlled Trial. J Behav Health Serv Res 39, 80–90. https://doi.org/10.1007/s11414-011-9249-8

Lin, K., Stubbs, B., Zou, W., Zheng, W., Lu, W., Gao, Y., Chen, K., Wang, S., Liu, J., Huang, Y., Guan, L., Wong, M.N.K., Wang, R., Lam, B.Y.-H., Xu, G., 2020. Aerobic exercise impacts the anterior cingulate cortex in adolescents with subthreshold mood syndromes: a randomized controlled trial study. Transl Psychiatry 10, 155. https://doi.org/10.1038/s41398-020-0840-8

Lin, L., Chu, H., Murad, M.H., Hong, C., Qu, Z., Cole, S.R., Chen, Y., 2018. Empirical Comparison of Publication Bias Tests in Meta-Analysis. J GEN INTERN MED 33, 1260–1267. https://doi.org/10.1007/s11606-018-4425-7

MacMahon, J.R., Gross, R.T., 1988. Physical and psychological effects of aerobic exercise in delinquent adolescent males. Am J Dis Child 142, 1361–1366. https://doi.org/10.1001/archpedi.1988.02150120115053

Mawdsley, D., Bennetts, M., Dias, S., Boucher, M., Welton, N.J., 2016. Model-Based Network Meta-Analysis: A Framework for Evidence Synthesis of Clinical Trial Data. CPT Pharmacometrics Syst Pharmacol 5, 393–401. https://doi.org/10.1002/psp4.12091

Mohammadi, M., Abhar, 2011. A study and comparison of the effect of team sports (soccer and volleyball) and individual sports (table tennis and badminton) on depression among high school students. Australian journal of basic and applied sciences 5, 1005–1011.

Nabkasorn, C., Miyai, N., Sootmongkol, A., Junprasert, S., Yamamoto, H., Arita, M., Miyashita, K., 2006. Effects of physical exercise on depression, neuroendocrine stress hormones and physiological fitness in adolescent females with depressive symptoms. European Journal of Public Health 16, 179–184. https://doi.org/10.1093/eurpub/cki159

Olive, L.S., Byrne, D., Cunningham, R.B., Telford, R.M., Telford, R.D., 2019. Can physical education improve the mental health of children? The LOOK study cluster-randomized controlled trial. Journal of Educational Psychology 111, 1331–1340. https://doi.org/10.1037/edu0000338

Pedder, H., Dias, S., Bennetts, M., Boucher, M., Welton, N.J., 2019. Modelling time-course relationships with multiple treatments: Model-based network meta-analysis for continuous summary outcomes. Res Synth Methods 10, 267–286. https://doi.org/10.1002/jrsm.1351

Petty, K.H., Davis, C.L., Tkacz, J., Young-Hyman, D., Waller, J.L., 2009. Exercise effects on depressive symptoms and self-worth in overweight children: a randomized controlled trial. J Pediatr Psychol 34, 929–939. https://doi.org/10.1093/jpepsy/jsp007

Romero-Pérez, E.M., González-Bernal, J.J., Soto-Cámara, R., González-Santos, J., Tánori-Tapia, J.M., Rodríguez-Fernández, P., Jiménez-Barrios, M., Márquez, S., de Paz, J.A., 2020. Influence of a Physical Exercise Program in the Anxiety and Depression in Children with Obesity. Int J Environ Res Public Health 17, 4655. https://doi.org/10.3390/ijerph17134655

Roth, D.L., Holmes, D.S., 1987. Influence of aerobic exercise training and relaxation training on physical and psychologic health following stressful life events. Psychosom Med 49, 355–365. https://doi.org/10.1097/00006842-198707000-00004

Rücker, G., Schwarzer, G., 2015. Ranking treatments in frequentist network meta-analysis works without resampling methods. BMC Medical Research Methodology 15, 58. https://doi.org/10.1186/s12874-015-0060-8

Salanti, G., Del Giovane, C., Chaimani, A., Caldwell, D.M., Higgins, J.P.T., 2014. Evaluating the quality of evidence from a network meta-analysis. PLoS One 9, e99682. https://doi.org/10.1371/journal.pone.0099682

Shachar, K., Ronen-Rosenbaum, T., Rosenbaum, M., Orkibi, H., Hamama, L., 2016. Reducing child aggression through sports intervention: The role of self-control skills and emotions. Children and Youth Services Review 71, 241–249. https://doi.org/10.1016/j.childyouth.2016.11.012

Shim, S.R., Lee, J., 2019. Dose-response meta-analysis: application and practice using the R software. Epidemiology and Health 41, e2019006. https://doi.org/10.4178/epih.e2019006

Silva, L.A.D., Doyenart, R., Henrique Salvan, P., Rodrigues, W., Felipe Lopes, J., Gomes, K., Thirupathi, A., Pinho, R.A.D., Silveira, P.C., 2020. Swimming training improves mental health parameters, cognition and motor coordination in children with Attention Deficit Hyperactivity Disorder. International Journal of Environmental Health Research 30, 584–592. https://doi.org/10.1080/09603123.2019.1612041

Talakoub, S., Gorbani, S., Hasanpour, M., Zolaktaf, V., Amini, M., 2012. Impact of exercise on affective responses in female adolescents with type I diabetes. Iran J Nurs Midwifery Res 17, 434–439.

Ter Veer, E., van Oijen, M.G.H., van Laarhoven, H.W.M., 2019. The Use of (Network) Meta-Analysis in Clinical Oncology. Front Oncol 9, 822. https://doi.org/10.3389/fonc.2019.00822

Van Valkenhoef, G., Dias, S., Ades, A.E., Welton, N.J., 2016. Automated generation of node-splitting models for assessment of inconsistency in network meta-analysis. Research Synthesis Methods 7, 80–93. https://doi.org/10.1002/jrsm.1167

Weintraub, D.L., Tirumalai, E.C., Haydel, K.F., Fujimoto, M., Fulton, J.E., Robinson, T.N., 2008. Team Sports for Overweight Children: The Stanford Sports to Prevent Obesity Randomized Trial (SPORT). Archives of Pediatrics & Adolescent Medicine 162, 232–237. https://doi.org/10.1001/archpediatrics.2007.43

Wheeler, D.C., Hickson, D.A., Waller, L.A., 2010. Assessing Local Model Adequacy in Bayesian Hierarchical Models Using the Partitioned Deviance Information Criterion. Comput Stat Data Anal 54, 1657–1671. https://doi.org/10.1016/j.csda.2010.01.025

White, I.R., Barrett, J.K., Jackson, D., Higgins, J.P.T., 2012. Consistency and inconsistency in network meta-analysis: model estimation using multivariate meta-regression. Research Synthesis Methods 3, 111–125. https://doi.org/10.1002/jrsm.1045

Williams, C.F., Bustamante, E.E., Waller, J.L., Davis, C.L., 2019. Exercise effects on quality of life, mood, and self-worth in overweight children: the SMART randomized controlled trial. Transl Behav Med 9, 451–459. https://doi.org/10.1093/tbm/ibz015

Yu, Y., Liu, S., Song, M., Fan, H., Zhang, L., 2020. Effect of Parent-Child Attachment on College Students’ Social Anxiety: A Moderated Mediation Model. Psychol Rep 123, 2196–2214. https://doi.org/10.1177/0033294119862981

Zhang, J., Ji, W., 2021. Exercise intervention improves the quality of life, anxiety, and depression of adolescent depression patients. Int J Clin Exp Med 14, 1292–1300.

Supplementary file 8. PRISMA NMA Checklist of Items to Include When Reporting A Systematic Review Involving a Network Meta-analysis

| Section/Topic | Item | Checklist Item | Reported in section/file |
| --- | --- | --- | --- |
| TITLE |  |  |  |
| Title | 1 | Identify the report as a systematic review incorporating a network meta-analysis (or related form of meta-analysis). | Title page |
| ABSTRACT |  |  |  |
| Structured summary | 2 | Provide a structured summary including, as applicable:  Background: main objectives  Methods: data sources; study eligibility criteria, participants, and interventions; study appraisal; and *synthesis methods, such as network meta-analysis.*  Results: number of studies and participants identified; summary estimates with corresponding confidence/credible intervals; treatment rankings may also be discussed. Authors may choose to summarize pairwise comparisons against a chosen treatment included in their analyses for brevity.  Discussion/Conclusions: limitations; conclusions and implications of findings.  Other: primary source of funding; systematic review registration number with registry name. | Abstract |
| INTRODUCTION |  |  |  |
| Rationale | 3 | Describe the rationale for the review in the context of what is already known*, including mention of why a network meta-analysis has been conducted.* | Introduction |
| Objectives | 4 | Provide an explicit statement of questions being addressed, with reference to participants, interventions, comparisons, outcomes, and study design (PICOS). | Introduction |
| METHODS |  |  |  |
| Protocol and registration | 5 | Indicate whether a review protocol exists and if and where it can be accessed (e.g., Web address); and, if available, provide registration information, including registration number. | Methods; PROSPERO  CRD42024596771. |
| Eligibility criteria | 6 | Specify study characteristics (e.g., PICOS, length of follow-up) and report characteristics (e.g., years considered, language, publication status) used as criteria for eligibility, giving rationale. *Clearly describe eligible treatments included in the treatment network, and note whether any have been clustered or merged into the same node (with justification).* | Methods (Section 2.2);  Supplementary file 4.1 |
| Information sources | 7 | Describe all information sources (e.g., databases with dates of coverage, contact with study authors to identify additional studies) in the search and date last searched. | Methods (Section 2.1) |
| Search | 8 | Present full electronic search strategy for at least one database, including any limits used, such that it could be repeated. | Supplementary file 1 |
| Study selection | 9 | State the process for selecting studies (i.e., screening, eligibility, included in systematic review, and, if applicable, included in the meta-analysis). | **Methods (Section 2.3)** |
| Data collection process | 10 | Describe method of data extraction from reports (e.g., piloted forms, independently, in duplicate) and any processes for obtaining and confirming data from investigators. | **Methods (Section 2.4)** |
| Data items | 11 | List and define all variables for which data were sought (e.g., PICOS, funding sources) and any assumptions and simplifications made. | Methods (Section 2.4);  Supplementary file 4.2 |
| Geometry of the network | S1 | Describe methods used to explore the geometry of the treatment network under study and potential biases related to it. This should include how the evidence base has been graphically summarized for presentation, and what characteristics were compiled and used to describe the evidence base to readers. | Results (Section 3.1);  Supplementary file 6.1 |
| Risk of bias within individual studies | 12 | Describe methods used for assessing risk of bias of individual studies (including specification of whether this was done at the study or outcome level), and how this information is to be used in any data synthesis. | Methods (Section 2.6) |
| Summary measures | 13 | State the principal summary measures (e.g., risk ratio, difference in means). Also describe the use of additional summary measures assessed, such as treatment rankings and surface under the cumulative ranking curve (SUCRA) values, as well as modified approaches used to present summary findings from meta-analyses. | Methods (Section 2.5);  Supplementary file 4.3 |
| Planned methods of analysis | 14 | Describe the methods of handling data and combining results of studies for each network meta-analysis. This should include, but not be limited to:   - Handling of multi-arm trials; - Selection of variance structure; - Selection of prior distributions in Bayesian analyses; and - Assessment of model fit. | Methods (Section 2.7);  Supplementary file 4.3 |
| Assessment of Inconsistency | S2 | Describe the statistical methods used to evaluate the agreement of direct and indirect evidence in the treatment network(s) studied. Describe efforts taken to address its presence when found. | Methods (Section 2.7);  Supplementary file 6.2–6.3 |
| Risk of bias across studies | 15 | Specify any assessment of risk of bias that may affect the cumulative evidence (e.g., publication bias, selective reporting within studies). | Methods (Section 2.6);  Results (Section 3.4)  Supplementary file 3; Supplementary file 5.2; Supplementary file 6.5 |
| Additional analyses | 16 | Describe methods of additional analyses if done, indicating which were pre-specified. This may include, but not be limited to, the following:   - Sensitivity or subgroup analyses; - Meta-regression analyses; - Alternative formulations of the treatment network; and - Use of alternative prior distributions for Bayesian analyses (if applicable). | Methods (Section 2.7);  Results (Section 3.7)  Supplementary file 6.4; Supplementary file 7.3 |
| RESULTS† |  |  |  |
| Study selection | 17 | Give numbers of studies screened, assessed for eligibility, and included in the review, with reasons for exclusions at each stage, ideally with a flow diagram. | Results (Section 3.1)  Figure 1 |
| Presentation of network structure | S3 | Provide a network graph of the included studies to enable visualization of the geometry of the treatment network. | Results (Section 3.1);  Supplementary file 6.1 |
| Summary of network geometry | S4 | Provide a brief overview of characteristics of the treatment network. This may include commentary on the abundance of trials and randomized patients for the different interventions and pairwise comparisons in the network, gaps of evidence in the treatment network, and potential biases reflected by the network structure. | Results (Section 3.2 and 3.5) |
| Study characteristics | 18 | For each study, present characteristics for which data were extracted (e.g., study size, PICOS, follow-up period) and provide the citations. | Results (Section 3.2)  Supplementary file 2 |
| Risk of bias within studies | 19 | Present data on risk of bias of each study and, if available, any outcome level assessment. | Results (Section 3.3)  Supplementary file 3 |
| Results of individual studies | 20 | For all outcomes considered (benefits or harms), present, for each study: 1) simple summary data for each intervention group, and 2) effect estimates and confidence intervals. *Modified approaches may be needed to deal with information from larger networks.* | Results (Section 3.2–3.7)  Supplementary file 2  Supplementary file 5-7 |
| Synthesis of results | 21 | Present results of each meta-analysis done, including confidence/credible intervals. In larger networks, authors may focus on comparisons versus a particular comparator (e.g. placebo or standard care), with full findings presented in an appendix. League tables and forest plots may be considered to summarize pairwise comparisons. If additional summary measures were explored (such as treatment rankings), these should also be presented. | Results (Section 3.4–3.7);  Figure 2–5;  Table 1;  Supplementary files 5–7 |
| Exploration for inconsistency | S5 | Describe results from investigations of inconsistency. This may include such information as measures of model fit to compare consistency and inconsistency models, *P* values from statistical tests, or summary of inconsistency estimates from different parts of the treatment network. | Results (Section 3.5);  Supplementary file 6.2; Supplementary file 6.3 |
| Risk of bias across studies | 22 | Present results of any assessment of risk of bias across studies for the evidence base being studied. | Results (Section 3.3–3.4);  Supplementary file 3; Supplementary file 5.2; Supplementary file 6.5 |
| Results of additional analyses | 23 | Give results of additional analyses, if done (e.g., sensitivity or subgroup analyses, meta-regression analyses*, alternative network geometries studied, alternative choice of prior distributions for Bayesian analyses,* and so forth). | Results (Section 3.5 and 3.7);  Supplementary file 6.4; Supplementary file 7.3 |
| DISCUSSION |  |  |  |
| Summary of evidence | 24 | Summarize the main findings, including the strength of evidence for each main outcome; consider their relevance to key groups (e.g., healthcare providers, users, and policy-makers). | Discussion (Sections 4.1–4.3) |
| Limitations | 25 | Discuss limitations at study and outcome level (e.g., risk of bias), and at review level (e.g., incomplete retrieval of identified research, reporting bias). *Comment on the validity of the assumptions, such as transitivity and consistency. Comment on any concerns regarding network geometry (e.g., avoidance of certain comparisons).* | Discussion (Sections 4.4) |
| Conclusions | 26 | Provide a general interpretation of the results in the context of other evidence, and implications for future research. | Conclusion |
| FUNDING |  |  |  |
| Funding | 27 | Describe sources of funding for the systematic review and other support (e.g., supply of data); role of funders for the systematic review. This should also include information regarding whether funding has been received from manufacturers of treatments in the network and/or whether some of the authors are content experts with professional conflicts of interest that could affect use of treatments in the network. | Funding |

PICOS = population, intervention, comparators, outcomes, study design.

* Text in italics indicates wording specific to reporting of network meta-analyses that has been added to guidance from the PRISMA statement.

† Authors may wish to plan for use of appendices to present all relevant information in full detail for items in this section.
